# CHOP favors endoplasmic reticulum stress-induced apoptosis in hepatocellular carcinoma cells via inhibition of autophagy

**DOI:** 10.1371/journal.pone.0183680

**Published:** 2017-08-25

**Authors:** Yan Lei, Shuiliang Wang, Bingshuang Ren, Jin Wang, Jin Chen, Jun Lu, Shihuai Zhan, Yunfeng Fu, Lianghu Huang, Jianming Tan

**Affiliations:** Department of Fujian Provincial Key Laboratory of Transplant Biology, Fuzhou General Hospital, Xiamen University, Fuzhou, Fujian, China; Duke University School of Medicine, UNITED STATES

## Abstract

C/EBP-homologous protein (CHOP) is an important component of the endoplasmic reticulum (ER) stress response. We demonstrated the induction of ER stress in response to tunicamycin stimulation, as evidenced by increased expression of chaperone proteins Grp78, Grp94, and enhanced eukaryotic initiation factor 2 subunit 1 (eIF2α) phosphorylation in hepatocellular carcinoma cells. Tunicamycin-induced ER stress resulted in apoptosis and autophagy simultaneously. While inhibition of autophagy mediated by 3-methyladenine pretreatment or direct knockdown of LC3B promoted cell apoptosis, activation of autophagy with rapamycin decreased tunicamycin- induced apoptosis in HCC cells. Furthermore, CHOP was shown to be significantly upregulated upon treatment with tunicamycin in HCC cells. Specific knockdown of CHOP not only enhanced tunicamycin-induced autophagy, but also significantly attenuated ER stress-induced apoptosis in HCC cells. Accordingly, simultaneous inhibition of autophagy in HCC cells with CHOP-knockdown could partially resensitize ER stress-induced apoptosis. Taken together, our data indicate that CHOP may favor ER stress-induced apoptosis in HCC cells via inhibition of autophagy *in vitro*.

## Introduction

Tumor hypoxia inhibits the formation of protein glycosylation and disulfide bonds, resulting in the accumulation of unfolded or misfolded proteins in endoplasmic reticulum (ER). This condition is defined as ER stress, which reflects an imbalance between the cellular demand for ER function and ER protein folding ability [1,2]. Prolonged or severe ER stress eventually results in cell apoptosis. Cellular adaptation to ER stress is achieved by the activation of a highly conserved signal transduction pathway known as the unfolded protein response (UPR)[3,4]. In tumors, the sensors of ER stress are PERK (PKR-like ER kinase; also known as eukaryotic translation initiation factor 2 alpha kinase 3 or EIF2AK3), ATF6 (activating transcription factor 6), and IRE1 (inositol-requiring enzyme 1). These 3 proteins activate the unfolded protein response. During ER stress, PERK dissociates from Grp78/BiP and activates itself by oligomerization and phosphorylation, which directly phosphorylates translation initiation factor eIF2α to attenuate general protein synthesis [5,6]. However, the activation of PERK also leads to increased translation of transcription factors such as ATF4, which promote transcription of genes related to cell survival, as well as pro-apoptotic factors such as CHOP (C/EBP homologous protein) [7–9]. CHOP can further downregulate the anti-apoptotic protein Bcl-2 and alter the redox state of the cell [10,11], thus sensitizing cells to apoptosis. Moreover, CHOP also promotes the expression of GADD45 (growth arrest and DNA-damage-inducible protein), triggering cell apoptosis by completely blocking protein synthesis [12].

Autophagy is a highly conserved system for the degradation of misfolded proteins and damaged organelles, and the recycling of amino acids for the synthesis of essential proteins [13,14]. Autophagy is controlled by a set of evolutionarily conserved autophagy-related proteins; its regulation involves more than 30 autophagy-related genes (ATG) in yeast, or 15 homologues in mammals [15]. It has been demonstrated by serial studies that autophagy has an important role in promoting cell survival under severe stress conditions [16–19]. Several lines of evidence also show that the eIF2α-ATF4 pathway contributes to the activation of autophagy that was induced by ER stress [20–23].

In cancer biology, autophagy is generally believed to act as a tumor suppressor in the early stages of cancer by protecting cells from oxidative stress and genomic instability [24,25]. However, during tumor progression, many cancers come to depend on autophagy as a source of nutrients[26].A study by B'chir *et al*[27]showed that CHOP both induces apoptosis and limits autophagy when amino acid starvation is prolonged.

In our current study, we investigated whether there is interplay between CHOP and autophagy in regulating ER stress-induced apoptosis in hepatocellular carcinoma (HCC) cells. We found that while tunicamycin (TM)-induced ER stress resulted in autophagy and apoptosis simultaneously, CHOP was evidenced to favor ER stress-induced apoptosis in HCC cells by inhibiting autophagy *in vitro*.

## Materials and methods

### Antibodies and reagents

Rapamycin (37094), 3-methyladenine (3-MA, M9281), and antibody against actin (a5441) were purchased from Sigma (Louis, MO, USA).Tunicamycin (11089-65-9) was purchased from Enzo Life Sciences (Madison Avenue, NY, USA). Antibodies against CHOP(L63f7), P-eIF2α (Ser51), BiP (C50B12), Bax (D3R2M), Caspase3 (8G10), Cleaved Caspase9 (Asp353), Cleaved PARP (Asp214), P-ULK1 (Ser737), LC3B (D11), Beclin-1 (D40C5), Atg5 (D5F5U), p62 (D5E2), eIF2α or Grp94 used in the western blot analysis were obtained from Cell Signaling Technology (Danvers, MA, USA). The Annexin-V-FLUOS Staining kit (Cat.No.11858777001) and cell proliferation reagent water-soluble terazoliumsalt (WST)-1 were purchased from Roche (Mannheim, Germany).

### Cell culture

Mouse HCC cells Hepa 1–6 were obtained from the cell bank of the Chinese Academy of Sciences (Shanghai, China) and maintained in high glucose Dulbecco's Modified Eagle's medium media (Hyclone, Logan, UT, USA) containing 100 U/mL penicillin/streptomycin and 10% fetal bovine serum (FBS) (Gibco, Gran Island, NY, USA). Human embryonic kidney cells HEK293T were maintained in DMEM/F12 medium supplemented with 10% FBS. All cell lines were cultured in a 37°C humidified atmosphere containing 95% air and 5% CO_2_ and were split twice per week.

### Construction of pLKO.1-CHOPshRNA-1, pLKO.1-CHOPshRNA-2 and pLKO.1-LC3B-shRNA

We employed the replication-incompetent lentiviral vector pLKO.1 (Sigma, Louis, MO, USA) chosen by the RNAi Consortium (TRC) for cloning and expressing short hairpin RNA (shRNA) sequences. A 1.9-kb stuffer in pLKO.1-TRC was released by digestion with restriction enzymes Age I and EcoR I. The synthesized DNA templates of shRNAs targeting CHOP or LC3B (Table 1) were annealed and then subcloned into the space between the Age I and EcoR I sites to construct pLKO.1-CHOPshRNA-1, pLKO.1-CHOPshRNA-2, and pLKO.1-LC3BshRNA, respectively. All 3 vectors were verified by direct DNA sequencing.

**Table 1:** Target sequences of shRNAs used in this study.

	Sense	Anti-sense
*CHOPshRNA 1*	5'-CCGGGAAACGAAGAGGAAGAATCAACTCGAGTTGATTCTTCCTCTTCGTTTCTTTTTG-3'	5'-AATTCAAAAAGAAACGAAGAGGAAGAATCAACTCGAGTTGATTCTTCCTCTTCGTTTC-3'
*CHOPshRNA 2*	5'-CCGGTGAAGAGAACGAGCGGCTCAACTCGAGTTGAGCCGCTCGTTCTCTTCATTTTTG-3'	5'-AATTCAAAAATGAAGAGAACGAGCGGCTCAACTCGAGTTGAGCCGCTCGTTCTCTTCA-3'
*MAP1LC3BshRNA*	5'-CCGGGCTCAATGCTAACCAAGCCTTCTCGAGAAGGCTTGGTTAGCATTGAGCTTTTTG-3'	5'-AATTCAAAAAGCTCAATGCTAACCAAGCCTTCTCGAGAAGGCTTGGTTAGCATTGAGC-3'

### Production of lentivirus

To produce lentivirus, the lentiviral expression vector pLKO.1-ConshRNA, pLKO.1-CHOPshRNA-1, pLKO.1-CHOPshRNA-2, or pLKO.1-LC3BshRNA and lentivirus packaging plasmids psPAX2 and pMD2.G were co-transfected into the virus packaging cell line HEK293T using PEIin accordance with the standard procedure. After 24 h, the culture media were changed for fresh DMEM/F12 media (10% FBS). The virus-containing media were collected, aliquoted, and stored at –80°C.

### Specific knockdown of CHOP or LC3B in Hepa 1–6 cells

To achieve specific knockdown of CHOP or LC3B in HCC cells, the lentivirus-containing media (800μL) were thawed completely at room temperature. Another 9.2 mLof fresh medium and hexadimethrine bromide (Polybrene; 8 μg/mL) was added into the virus-containing media. Then the culture media of the Hepa 1–6 cells was replaced with the lentivirus-containing media. After 24 h, the virus-infected cells were selected with puromycin (1 μg/mL) for an additional 10days to establish sublines of Hepa 1–6 cells with stable knockdown of CHOP or LC3B. Those cells were then collected and subjected to the required experiments.

### Cell survival assay

Hepa 1–6 cells were seeded in 96-well plates and cultured in medium (10%FBS) with or without rapamycin, 3-MA, TM for 24h. The viability of the cells was measured using a WST-1 cell viability kit in accordance with the manufacturer's instructions. Briefly, WST-1 was added to each well, and the cells were incubated for 2h. The plates were shaken thoroughly for 1 min, and the absorbance of the samples measured at 450nm. The experiment was repeated 3 times.

### Real-time quantitative reverse transcription-PCR (qRT-PCR)

Total RNA was extracted from cells using the total RNA kit (Omega Bio-Tek, Norcross, GA, USA), and cDNAs was synthesized using a Revert Aid First Strand cDNA Synthesis Kit (Fermentas, Waltham, MA, USA) in accordance with the manufacturer's instructions. The primer sequences used for qRT-PCR analysis are listed in the Table 2. Real-time quantitative RT-PCR was performed with a ABI PRISM 7900 sequence detection system (Los Altos, CA, USA) using SYBR Green PCR Master Mix (Roche, Mannheim, Germany).Thermal cycling conditions were:95°C for 10 min; 95°C for 15 s and then 58°C for 30 s for 40 cycles. The relative amount of mRNA of each gene in each sample was calculated using the 2^−ΔΔCT^ method. The expression of β-actin was used as an internal control for all qRT-PCR.

**Table 2:** List of primers used for quantitative RT-PCR analysis.

Gene	Forward sequence(5'→3')	Reverse sequence(5'→3')
*Grp94*	TTGTGGCCAGTCAGTATGGA	TGAGGCGAAGCATTCTTTCT
*Grp78*	GTTCCGCTCTACCATGAAGC	GGGGACAAACATCAAGCAGT
*CHOP*	TATCTCATCCCCAGGAAACG	CTGCTCCTTCTCCTTCATGC
*β-actin*	GGGAATGGGTCAGAAGGACT	GGGGTGTTGAAGGTCTCAAA

### Immunofluorescence assay

Hepa 1–6 cells were seeded in a 24-well plate with 3×10^3^ cells per well for overnight and then incubated with 0.8 μg/mL tunicamycin for additional 12 or 24 h. Cells were fixed with 4% paraformaldehyde for 30 min after washing with PBS for 3 times. After that, fixed cells were then incubated with 0.4% Triton-X 100 and 4% BSA in PBS for 2 h at room temperature following by incubation with specific primary antibodies against LC3B (1:200, D11, Cell Signaling Technology) at 4°C for overnight. After washing with PBS, cells were incubated with anti-Rabbit IgG (H+L) secondary antibody for 1 h and then incubated with DAPI (1 mg/mL) for 5 minutes at room temperature. Images were obtained using fluorescence microscope (Olympus, Japan).

### Flow cytometry assays

Hepa 1–6 cells were incubated in the absence or presence of 1μg/mLTM for 24 h. The cells were harvested by 0.25% trypsin, washed twice with cold phosphate buffered saline (PBS), and then resuspended in 500μL of buffer containing 50 μg/mLpropidiumiodide (PI), followed by incubation for 5 min at room temperature. The cells were finally analyzed with a FACSort flow cytometer (Becton-Dickinson, Franklin, NJ, USA).

### Western blot analysis

Hepa 1–6 cells were washed in ice-cold PBS twice, and lysed in buffer with protease inhibitor and phosphatase inhibitor, and then centrifuged at 13000×g for 25 min at 4°C. The supernatant was collected and total proteins were quantified usingbicinchoninic acid (Pierce, Rockford, AL, USA,) method. The protein samples were loaded onto polyacrylamide gel and subjected to sodium dodecyl sulfate polyacrylamide gel electrophoresis (SDS-PAGE). Proteins were then transferred onto a polyvinylidenedifluoride (PVDF) membrane. The membrane was blocked with Tris-buffered saline and Tween 20 (TBST) containing 4% BSA for 1 h at room temperature. The membranes were incubated serially with primary antibodies at 4°C overnight. After washing with TBST 3 times for 8 min each, the membranes were incubated with secondary antibodies for 1–2 h at room temperature. The density of the corresponding bands was measured quantitatively using image analysis software (Bio-Red, Hercules, CA, USA) and corrected by reference to the value of β-actin.

### Acridine orange staining

Hepa 1–6 cells were stained with acridine orange solution (100μg/mL) for 10 min at 37°C, and washed with PBS twice. The cells were finally analyzed with a FACSort flow cytometer (Becton-Dickinson, Franklin, NJ, USA).

### Statistical analysis

Statistical analyses were performed using the SPSS software package (version 16.0; SPSS, Chicago, IL, USA). Quantitative data are presented as the mean ± standard deviation (SD). Data were analyzed using Student's *t*-test or ANOVA with dunnett’s *t*-test to evaluate inter-group differences. *P*<0.05 was considered as statistically significant.

## Results

### TM-induced ER stress results in apoptosis in HCC cells

**Fig 1:**
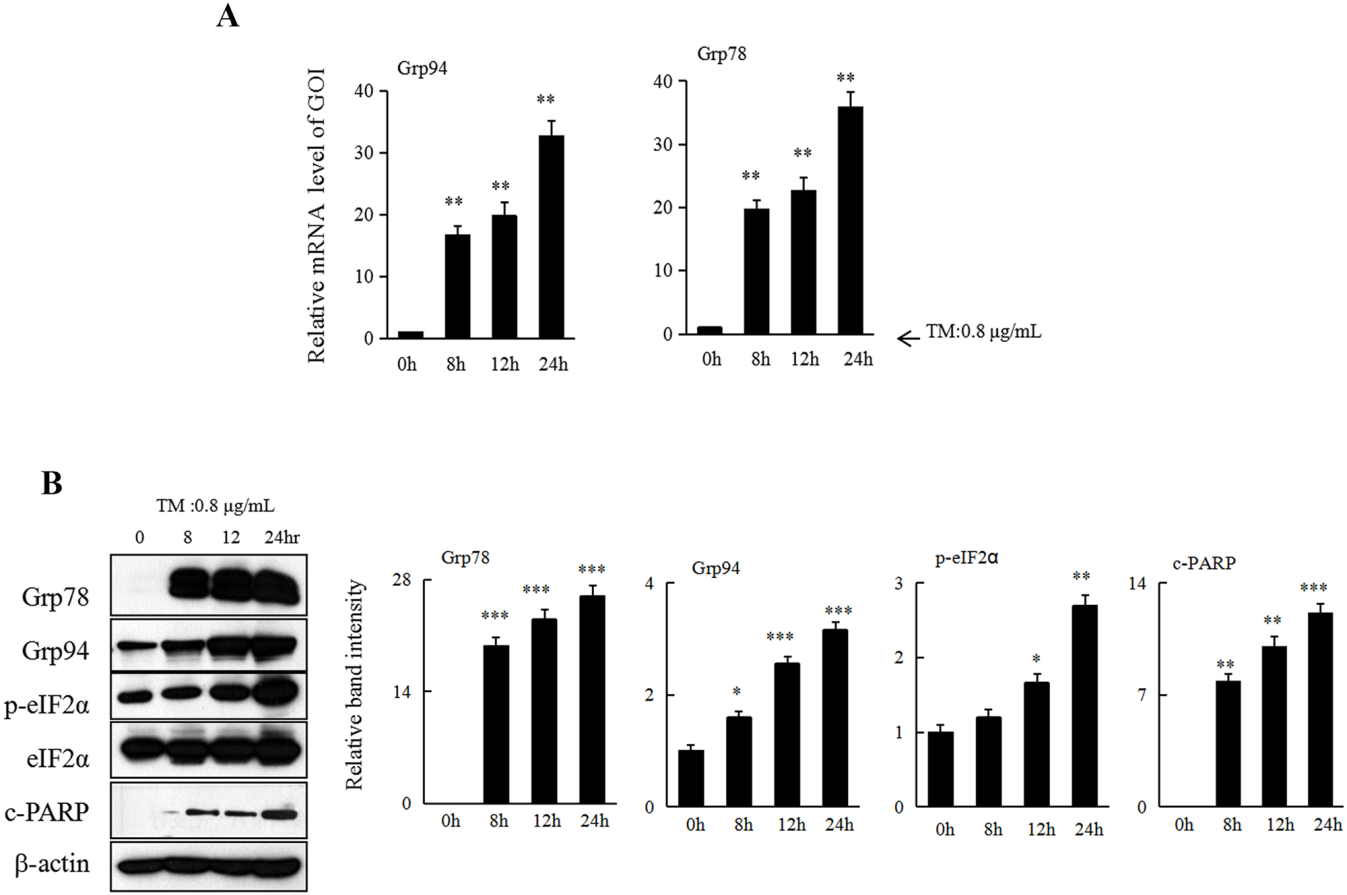
Tunicamycin(TM) induces ER stress, which in turn results in apoptosis of HCC cells. (A) Hepa 1–6 cells were treated with 0.8 μg/mL of TM for 0, 8, 12, or 24 h. Cells were collected and subjected to total RNA extraction. The mRNA levels of ER stressrelated genes Grp94 and Grp78 were measured by quantitative RT-PCR. All results were normalized with the internal control β-actin. Bars, SD. Data represent3 independent experiments.(B) Hepa 1–6 cells underwent aforementioned treatment were collected and subjected to western blot analyses with specific antibodies directed against Grp78, Grp94, p-eIF2α, eIF2α, c-PARP, or β-actin. The density of the corresponding bands was measured quantitatively using image analysis software and corrected by reference to the value of β-actin. Each data point is the mean ± SD of 3 independent experiments, *P<0.05 or **P<0.01 denotes significant difference from normal control Hepa1-6 cells.

As a well-known ER stressor, TM was firstly applied to treat HCC Hepa 1–6 cells. The expression of ER stress-related proteins Grp94 andGrp78 at mRNA levels were significantly increased in Hepa 1–6 cells upon treatment with TM (0.8μg/mL) (Fig 1A). In parallel with the changes in mRNAs, our western blot analysis showed that the expression of Grp94, Grp78, and p-eIF2α also markedly increased in those cells subjected to TM for 24 h (Fig 1B). Meanwhile, TM induced significant apoptosis in Hepa 1–6 cells as well as evidenced by increased cleaved PARP (c-PARP) (Fig 1B). Thus, our data here indicated that ER stress induced by TM could result in apoptosis in HCC cells.

### TM-induced ER stress induces autophagy in HCC cells in a time-dependent manner

**Fig 2:**
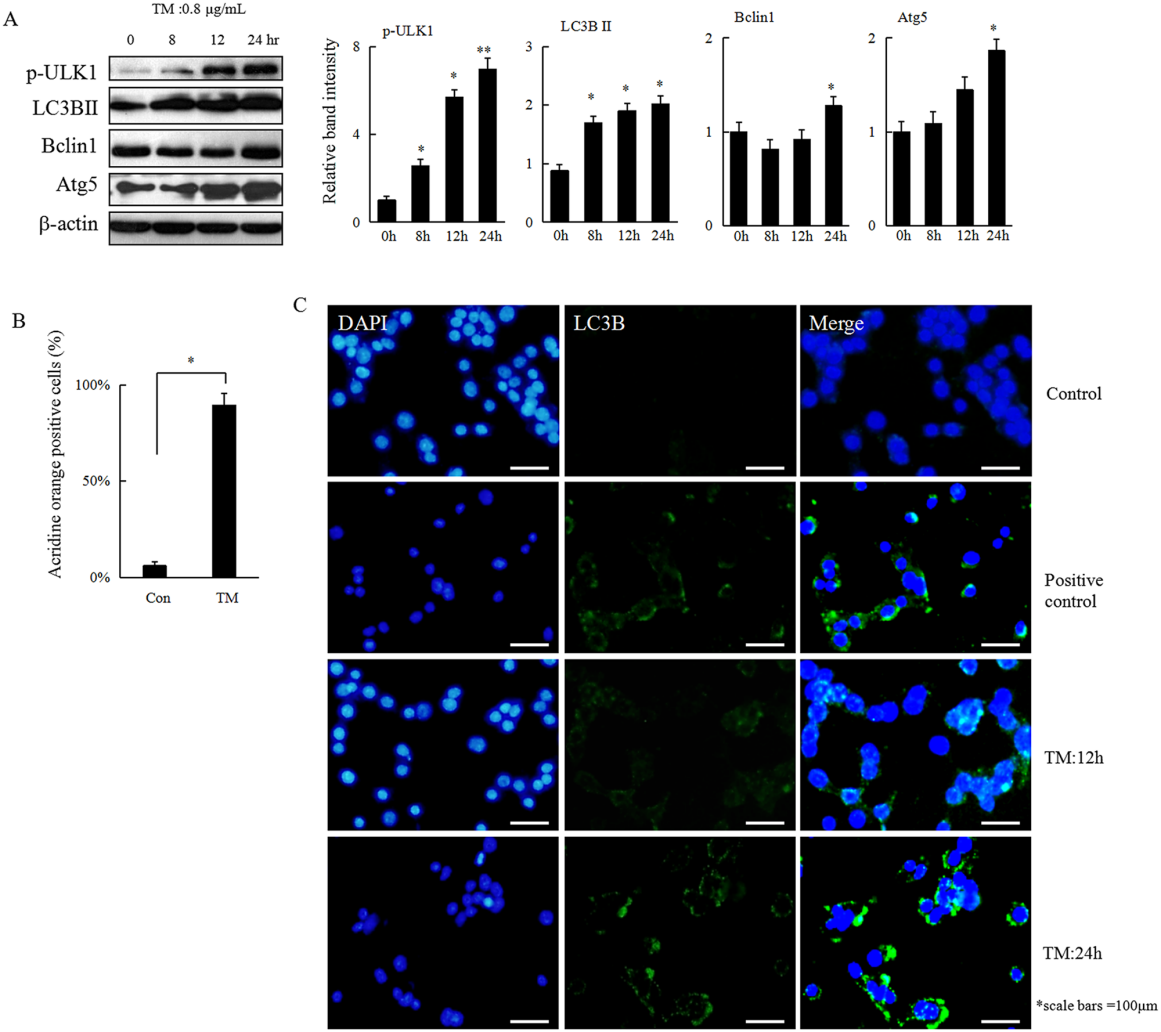
Autophagy is induced in response to ER stress in HCC cells. (A) Heap 1–6 cells were treated with TM (0.8 μg/mL) for 0, 8, 12, or 24 h. Cells were collected and subjected to western blot analyses with specific antibodies directed against p-ULK1, LC3B, Beclin1, Atg5, or β-actin. The density of the corresponding bands was measured quantitatively with an aforementioned method. Each data point is the mean ± SD of 3 independent experiments, *P<0.05 or **P<0.01 denotes a significant difference compared with normal control Hepa 1–6 cells. (B) Hepa1-6 cells were treated with TM (0.8 μg/mL) for 24 h and stained with acridine orange (1 mg/mL) for 15 min, followed immediately by detection using flow cytometry. The values were expressed as mean ± SD of 3 independent experiments, *P<0.05 denotes significant difference from normal control Hepa 1–6 cells. (C) Hepa 1–6 cells were treated with TM (0.8 μg/mL) for 12 h or 24 h and harvested for immunofluorescence assay and representative results are shown. Hepa 1–6 cells without or with strvation for 24 h were served as control or positive control, respectively.

Being a highly evolutionary conserved process, autophagy acts as a survival mechanism in cells exposed to stress. To investigate whether ER stress could also induce autophagy in HCC cells, Hepa 1–6 cells were treated with 0.8 μg/mL of TM for 0, 8, 12, or 24 h. LC3 II is considered an accurate marker of autophagy. Conversion of the cytosolic form LC3 I to its lipidated membrane-bound form LC3 II increased upon treatment with TM in a time-dependent manner (Fig 2A). Similar results were observed through acridine orange staining or immunofluorscence assay, which further demonstrated that autophagy was induced in HCC cells that are under ER stress (Fig 2B and 2C).

### Alteration of ER stress-induced autophagy regulates apoptosis in HCC cells

**Fig 3:**
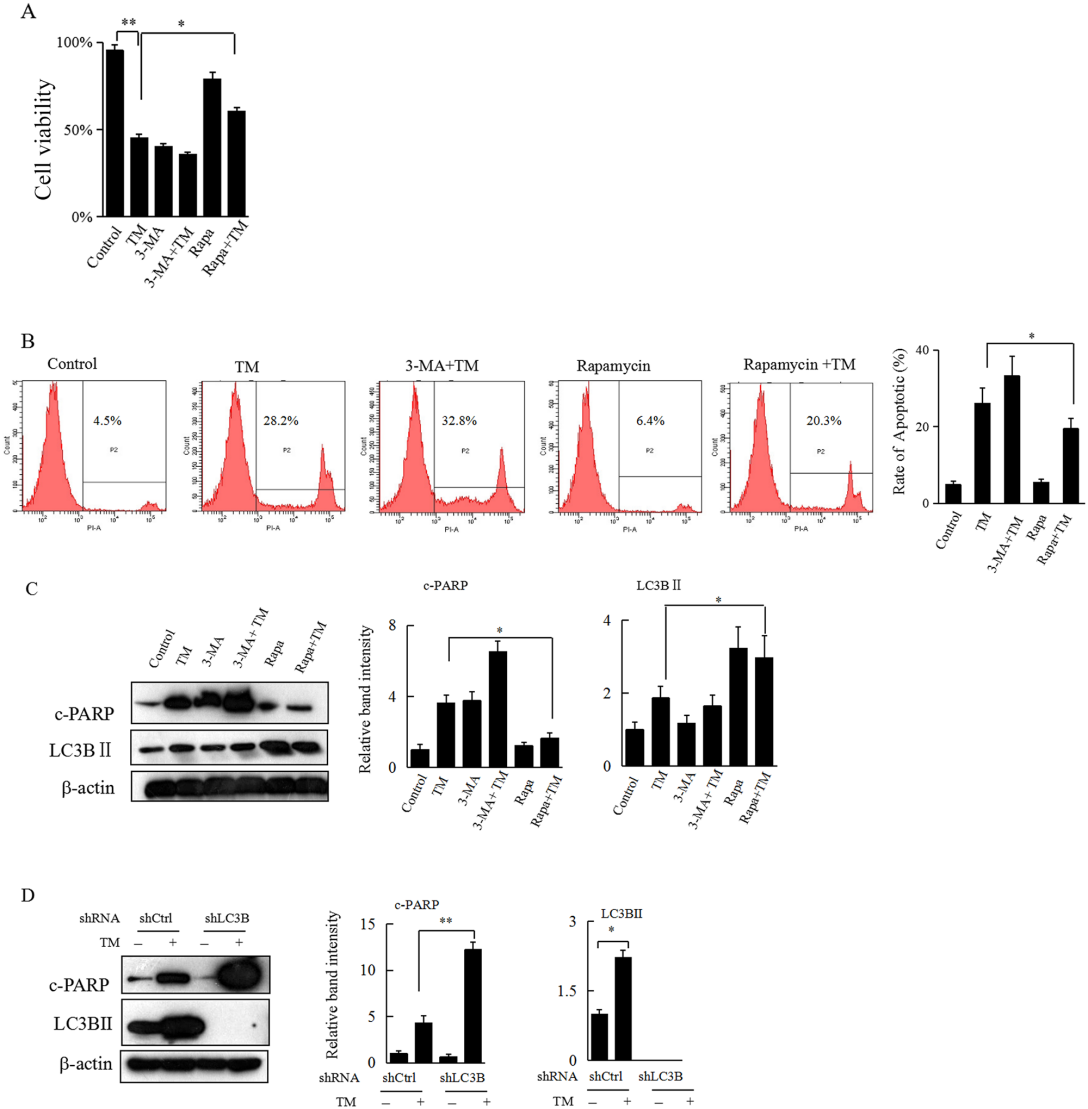
Inhibition of ER stress-induced autophagy aggravates, while activation of ER stress-induced autophagy decreases apoptosis in HCC cells. (A) The Hepa 1–6 cells were incubated with rapamycin (100 nM) or 3-MA (5 mM) for 2 h, followed by treatment with TM (0.8 μg/mL) for an additional 24 h. Cells were then subjected to WST-1 assay. Data represent the mean ± SD of 3 separate experiments. *P<0.05 or **P<0.01 denotes a significant difference from the indicated control. (B) Hepa 1–6 cells that underwent the same treatment as in Figure 3A were subjected to flow cytometry assay and representative results are shown. The rate of apoptosis in each group was calculated based on PI staining assays. (C) Hepa 1–6 cells that underwent the same treatment as in Figure 3A were collected and subjected to western blot analyses with specific antibodies directed against c-PARP, LC3B, or β-actin. The density of the corresponding bands was measured quantitatively with an aforementioned method. Each data point is the mean ± SD of 3 independent experiments, *P<0.05 or **P<0.01 denotes a significant difference from the indicated control. (D) Hepa 1–6 cells with or without specific knockdown of LC3B were treated with TM (0.8 μg/mL) for 24 h and then harvested for western blot analyses with specific antibodies directed against c-PARP, LC3B II, or β-actin.The density of the corresponding bands was measured quantitatively with an aforementioned method. *P<0.05 or **P<0.01 denotes a significant difference from the indicated control.

Having found that TM-induced ER stress results in significant apoptosis and autophagy in HCC cells, we then explored whether alteration of ER stress-induced autophagy influences apoptosis in HCC cells. The Hepa 1–6 cells were pretreated with autophagy activator rapamycin or autophagy inhibitor 3-MA for 2 h, followed by treatment with TM (0.8 μg/mL) for an additional 24 h. In the cell proliferation WST-1 assay, although pretreatment with 3-MA didn’t show any effect on TM-mediated growth inhibition of Hepa 1–6 cells, the inhibitory effect of TM on cell growth was significantly decreased upon pretreatment with rapamycin (Fig 3A). Both the flow cytometry assay and western blot analysis also revealed that pretreatment with 3-MA or rapamycin could simultaneously aggravate or decrease TM-induced apoptosis in Hepa 1–6 cells, respectively (Fig 3B and 3C). In addition, the inhibitor or activator effect of 3-MA or rapamycin on autophagy was verified by western blot analysis, as evidenced by decreased or increased expression of LC3 II, respectively (Fig 3C).Furthermore, direct knockdown of LC3B in Hepa 1–6 cells significantly potentiated TM-induced apoptosis (Fig 3D). Altogether, these results suggested that specific alteration of ER stress-induced autophagy could regulate apoptosis in HCC cells.

### Specific knockdown of CHOP attenuates ER stress-induced apoptosis in HCC cells

**Fig 4:**
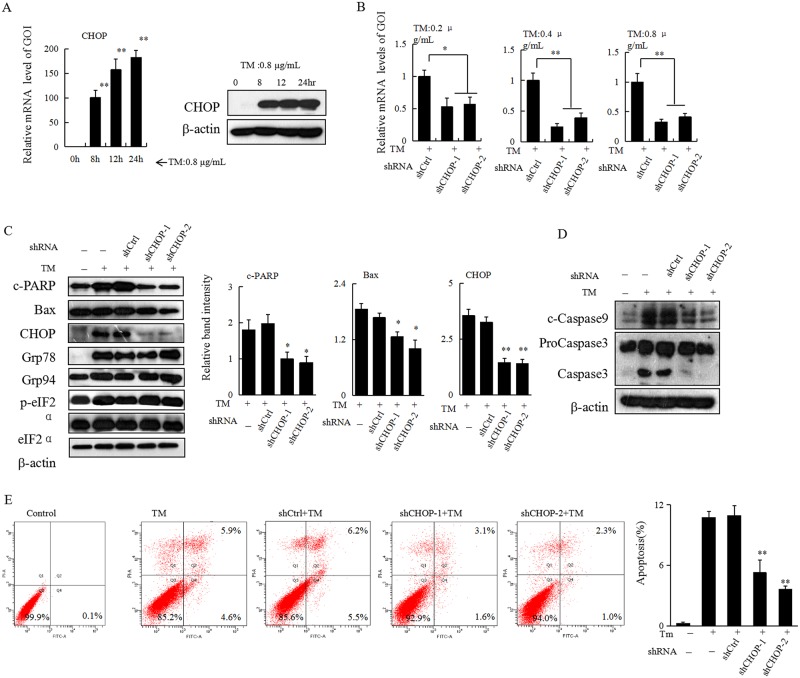
Knockdown of CHOP reduces TM-induced apoptosis in HCC cells. (A) Hepa 1–6 cells were treated with 0.8 μg/mL of TM for 0, 8, 12, or 24 h. Cells were collected and subjected to total RNA extraction. The mRNA levels of CHOP were measured by quantitative RT-PCR. All results were normalized with the internal control β-actin. Bars, SD. Data show the representative of 3 independent experiments. Meanwhile Hepa 1–6 cells given the aforementioned treatment were also collected and subjected to western blot analyses with specific antibodies directed against CHOP or β-actin. (B) Hepa 1–6 cells with or without knockdown of CHOP were treated with different doses of TM as indicated for 24 h. Cells were collected and subjected to total RNA extraction. The mRNA expression levels of CHOP were measured by RT-qPCR assays. All results were normalized with the internal control, β-actin. Bars, SD. The data are representative of 3 independent experiments. (C) Hepa 1–6 cells with or without knockdown of CHOP were treated with TM (0.8 μg/mL) for 24 h. Cells were collected and subjected to western blot analyses with specific antibodies directed against Grp94, Grp78, p-eIF2α, c-PARP, Bax, CHOP, or β-actin. The density of the corresponding bands was measured quantitatively with an aforementioned method. Each data point is the mean ± SD of 3 independent experiments, *P<0.05 or **P<0.01 denotes significant difference from TM-treated Hepa 1–6 cells with normal level of CHOP. (D) Hepa 1–6 cells underwent the same treatment as in Figure 4C were subjected to western blot analysis with specific antibodies directed against c-Caspase9, Caspase3, or β-actin. (E) Hepa 1–6 cells underwent the same treatment as in Figure 4C were subjected to flow cytometry assay and representative results are shown. The rate of apoptosis in each group was calculated based on Annexin V and PI staining assays. Representative experiments were carried out at least three times, **P<0.01 denotes significant difference from shCtrl group.

CHOP has been reported to have an important role in regulating cell death after ER stress. Since treatment with TM significantly induced both mRNA and protein expression of CHOP in Heap 1–6 cells (Fig 4A), to figure out whether CHOP also influences ER stress-induced apoptosis in HCC cells, a self-prepared lentiviral system was used to achieve a specific knockdown of CHOP in Hepa 1–6 cells. Compared with the control (shCtrl), both shCHOP-1 and shCHOP-2 exhibited specific knockdown effect of CHOP both in mRNA (Fig 4B) and protein levels (Fig 4C) in TM-treated Hepa 1–6 cells. Interestingly, while CHOP knockdown had no significant influence on the expression of ER stress-related proteins, including Grp94, Grp78, p-eIF2α, the expression of TM-induced cleaved-PARP, cleaved-caspase9, or cleaved-caspase3 was significantly reduced (Fig 4C and 4D). The flow cytometry assay further revealed that TM-induced apoptosis was significantly attenuated in Hepa 1–6 cells with downregulation of CHOP (Fig 4E). Taken together, our current data suggested that CHOP may have an important role in the regulation of ER stress-induced apoptosis.

### Role of CHOP in regulating apoptosis of HCC cells underwent prolonged ER stress

**Fig 5:**
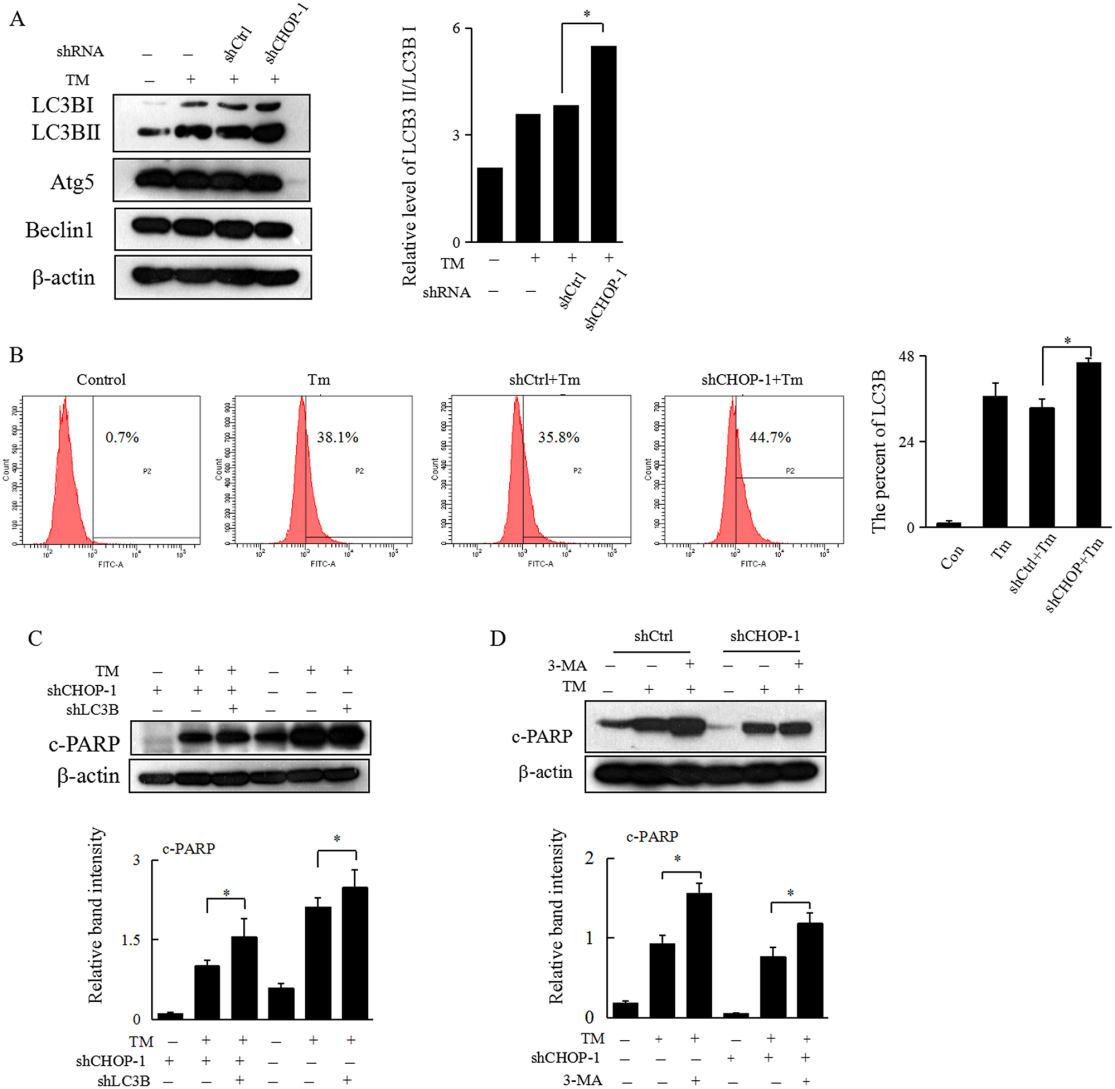
Downregulation of CHOP decreases apoptosis by activation of ER stress-induced autophagy. (A) Hepa 1–6 cells with or without knockdown of CHOP were treated with TM (0.8 μg/mL) for 24 h. Cells were collected and subjected to western blot analyses with specific antibodies directed against LC3B, Atg5, Beclin1, or β-actin. The density of the corresponding bands was measured quantitatively with an aforementioned method. Each datapoint is the mean ± SD of 3 independent experiments, *P<0.05 denotes significant difference from TM-treated Hepa 1–6 cells with normal levels of CHOP. (B) Hepa 1–6 cells underwent the same treatment as in Figure 5A were subjected to flow cytometry assay to detect the expression of LC3B and representative results are shown. Representative experiments were carried out at least three times, **P<0.01 denotes significant difference from shCtrl group. (C) Hepa 1–6 cells without or with knockdown of CHOP alone, or combined knockdown of CHOP and LC3B, were treated with TM (0.8 μg/mL) for 24 h. Cells were collected and subjected to western blot analyses with specific antibodies directed against c-PARP or β-actin. The density of the corresponding bands was measured quantitatively with an aforementioned method. Each data point is the mean ± SD of 3 independent experiments, *P<0.05 denotes significant difference from normal control Hepa 1–6 cells. (D) Hepa 1–6 cells with or without knockdown of CHOP were treatedt with TM (0.8 μg/mL) for 24 h, or 3-MA for 2 h, and then with TM (0.8 μg/mL) for an additional 24 h and then harvested for western blot analyses with specific antibodies directed against c-PARP or β-actin. *P<0.05 or **P<0.01 denotes significant difference compared to the TM-treated cells.

Previously we found that activation of autophagy decreases TM-induced apoptosis in Hepa 1–6 cells, while specific knockdown of CHOP also shows similar effect in regulating ER stress-induced apoptosis. We then hypothesized that CHOP may regulate apoptosis of HCC cells underwent prolonged ER stress through modulation of ER stress-induced autophagy. Interestingly, specific knockdown of CHOP did significantly enhance TM-induced autophagy, as evidenced by both western blot analysis and flow cytometry assay (Fig 5A and 5B). To further confirm our hypothesis here, autophagy was specificly inhibited via direct knockdown of LC3B or treatment with 3-MA in HCC cells with downregulation of CHOP. While specific knockdown of CHOP alone decreased ER stress-induced apoptosis in HCC cells, simultaneous inhibition of autophagy could partially resensitize ER stress-induced apoptosis. This suggested that downregulation of CHOP may attenuate ER stress-induced apoptosis via activation of autophagy in HCC cells (Fig 5C and 5D).

## Discussion

Accumulation of unfolded or misfolded proteins in the ER induces ER stress. In response to ER stress, chaperone molecules dissociate from the ER membrane to reduce protein synthesis, facilitate protein folding, and increase degradation of unfolded proteins [1,5,6].We demonstrate here the induction of ER stress in response to TM stimulation, as evidenced by increased expression of chaperone proteins Grp78 andGrp94 and enhanced eIF2α phosphorylation.

Autophagy functions to maintain energy homeostasis by degradation and recycling of damaged or harmful intracellular components [13,28].Overload of misfolded or unfolded proteins in the ER leads to a failure of protein degradation by proteasome, causing the upregulation of autophagy[29].In our current study, we further showed that autophagy was activated under TM-induced ER stress. More interestingly, while inhibition of autophagy mediated by 3-methyladenine pretreatment or direct knockdown of LC3B promoted cell apoptosis, activation of autophagy with rapamycin decreased TM-induced apoptosis in HCC cells. These data indicate that activation of autophagy may protect HCC cells from ER stress-induced damage to a certain extent.

**Fig 6:**
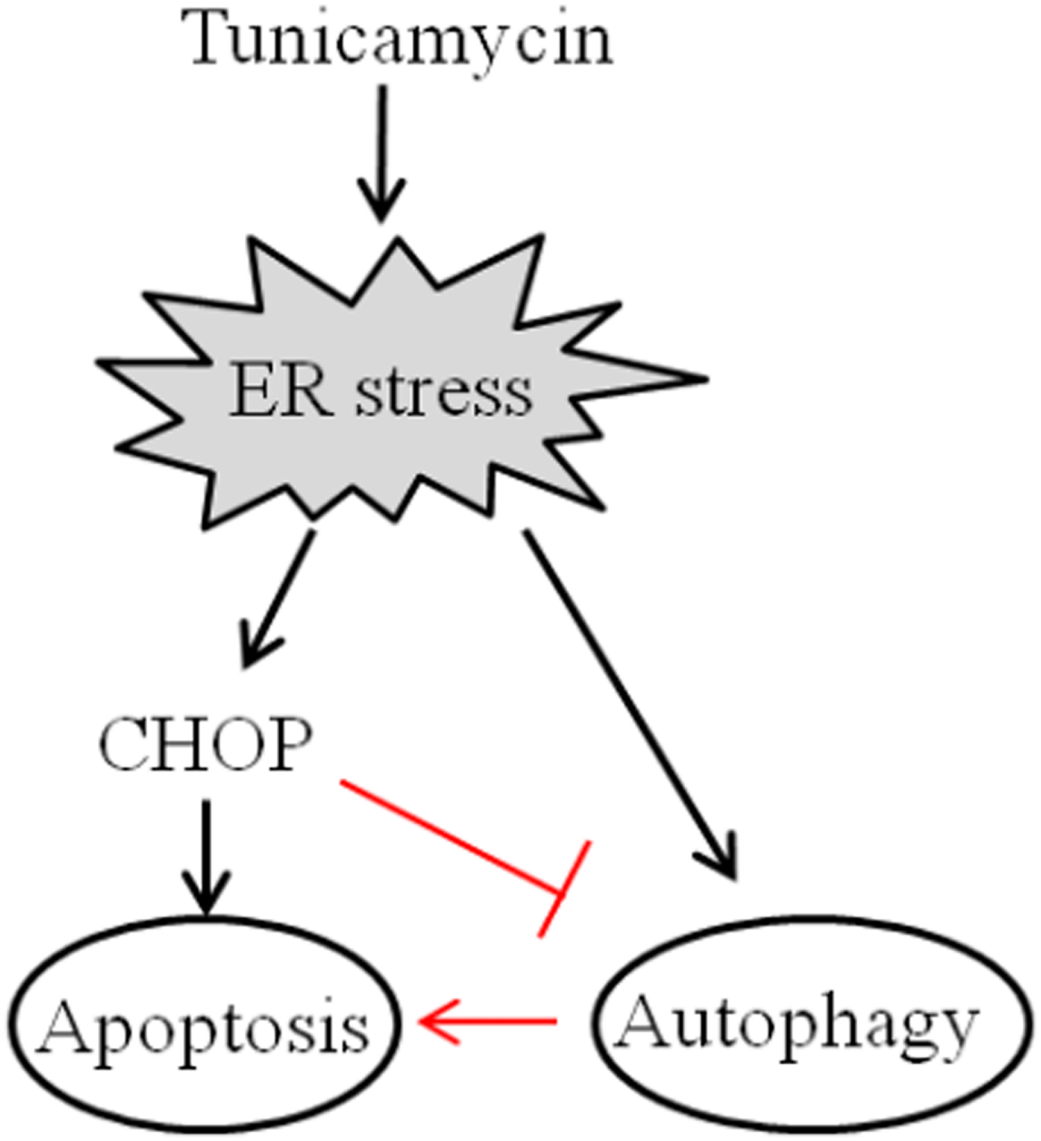
Model for CHOP regulation of autophagy and ER stress-induced apoptosis in HCC cells. TM-induced ER stress resulted in apoptosis as well as autophagy simultaneously in HCC cells (black line).CHOP may promote ER stress-induced apoptosis in HCC cells via inhibition of autophagy (red line).

CHOP is ubiquitously expressed at very low levels, but is strongly expressed in most cells when subjected to severe stress[30].Our present study also showed that upon treatment with TM, the expression of CHOP in HCC cells was significantly elevated at both mRNA and protein levels. Since TM induced-ER stress resulted in significant apoptosis simultaneously, to unravel further the possible influence of CHOP on ER stress-induced apoptosis in HCC cells, in the present study a self-prepared lentiviral system was used to achieve a specific knockdown of CHOP. While undergoing ER stress, specific knockdown of CHOP not only enhanced TM-induced autophagy, but also significantly attenuated ER stress-induced apoptosis in HCC cells. Most importantly, simultaneous inhibition of autophagy in HCC cells with downregulation of CHOP could partially resensitize ER stress-induced apoptosis. Thus, our data support the notion that CHOP may favor ER stress-induced apoptosis in HCC cells through not only its direct action in regulating pro-apoptotic proteins such as Bcl-2[10,11], but also inhibition of autophagy (summarized in Fig 6).

In conclusion, in the present study we present evidence that CHOP may favor ER stress-induced apoptosis in HCC cells by inhibiting autophagy *in vitro*. Our data suggest that there exists a complex interplay between CHOP and autophagy in HCC cells undergoing ER stress, although the underlying mechanism of how CHOP interacts with LC3B awaits further investigation.

## Supporting information

S1 Fig Representative results of WB using LC3B antibody pasted by Cell Signaling Technology. (JPG)

S2 Fig TM induced autophagy and apoptosis in a dose-dependent manner. Heap 1–6 cells were treated with TM (0.2, 0.4, 0.8, or 1.0 μg/mL) for 24 h and collected for western blot analyses with specific antibodies directed against LC3B, Caspase3, or β-actin.
(JPG)

S3 Fig qRT-PCR results of mRNA expression of Atg5, Beclin1, or LC3B. Hepa 1–6 cells were treated with TM (0.8 μg/mL) for 0, 8, 12, 16, or 24 h. Cells were collected and subjected to total RNA extraction. The relative mRNA levels of autophagy related genes Atg5, Beclin1, or LC3B were analyzed by quantitative RT-PCR.
(JPG)

S4 Fig Downregulation of CHOP increased the expression of LC3B. Hepa 1–6 cells with or without knockdown of CHOP were treated with TM (0.8 μg/mL) for 24 h. Cells were collected and subjected to western blot analyses with specific antibodies against LC3B, Atg5, Beclin1, or β-actin.
(JPG)

S5 Fig Downregulation of CHOP decreases apoptosis by activation of ER stress-induced Autophagy. (A) (B) We repeated the experiments that Hepa 1–6 cells without or with knockdown of CHOP alone, or combined knockdown of CHOP and LC3B, were treated with TM (0.8 μg/mL) for 24 h. Cells were collected and subjected to western blot analyses with specific antibodies directed against c-PARP or β-actin. (C) (D). We repeated the experiments that Hepa 1–6 cells with or without knockdown of CHOP were treated with TM (0.8 μg/mL) for 24 h, or 3-MA for 2 h, and then with TM (0.8 μg/mL) for an additional 24 h and then harvested for western blot analyses with specific antibodies directed against c-PARP or β-actin.
(JPG)

S6 Fig The induction of LCB3-II by treatment with TM was shown in a time-dependent manner. Heap 1–6 cells were treated with TM (0.8 μg/mL) for 0, 8, 12, or 24 h. Cells were subjected to western blot analyses with specific antibodies directed against LC3B or β-actin.
(JPG)

S7 Fig The induction of cell apoptosis by treatment with TM was shown in a time-dependent manner. Heap 1–6 cells were treated with TM (0.8 μg/mL) for 0, 8, 12, or 24 h. Cells were subjected to western blot analyses with specific antibodies directed against Caspase9, Caspase3 or β-actin.
(JPG)

S8 Fig The cell cycle analysis by flow cytometry assay. Heap 1–6 cells incubated either in control or TM (0.8 μg/mL) for 8, 12, or 24 h were stained with PI and detected by flow cytometry assay, and then analysis with Modifit.
(JPG)

S9 Fig Repaet data of Fig 1B. (JPG)

S10 Fig Repaet data of Fig 2A. (JPG)

S11 Fig Repaet data of Fig 3C. (JPG)

S12 Fig Repaet data of Fig 3D. (JPG)

S13 Fig Repaet data of Fig 4A. (JPG)

S14 Fig Repaet data of Fig 4C. (JPG)

S15 Fig Repaet data of Fig 5A. (JPG)

S16 Fig Raw data related to Fig 1B (c-PARP). (JPG)

S17 Fig Raw data related to Fig 4C (both CHOP and Grp94). (JPG)

S18 Fig Raw data related to Fig 5A (LC3B). (JPG)

S19 Fig Raw data related to S9 Fig (c-PARP left panel). (JPG)

S20 Fig Raw data related to S9 Fig (c-PARP right panel). (JPG)

S21 Fig Raw data related to S14 Fig (CHOP&Grp94 left panel). (JPG)

S22 Fig Raw data related to S14 Fig (CHOP&Grp94 right panel). (JPG)

S23 Fig Raw data related to S15 Fig (LC3B left panel). (JPG)

S24 Fig Raw data related to S15 Fig (LC3B right panel). (JPG)

S1 Table List of primers used for quantitative RT-PCR analysis. (JPG)

## Abbreviations

CHOP: C/EBP-homologous protein
ER stress: endoplasmic reticulum stress
HCC cells: hepatocellular carcinoma cells
