# Supplementary figures and images for: CHOP favors endoplasmic reticulum stress-induced apoptosis in hepatocellular carcinoma cells via inhibition of autophagy
Source: PLoS One. 2017 Aug 25;12(8):e0183680. doi: 10.1371/journal.pone.0183680 (PMC5571976; doi:10.1371/journal.pone.0183680)

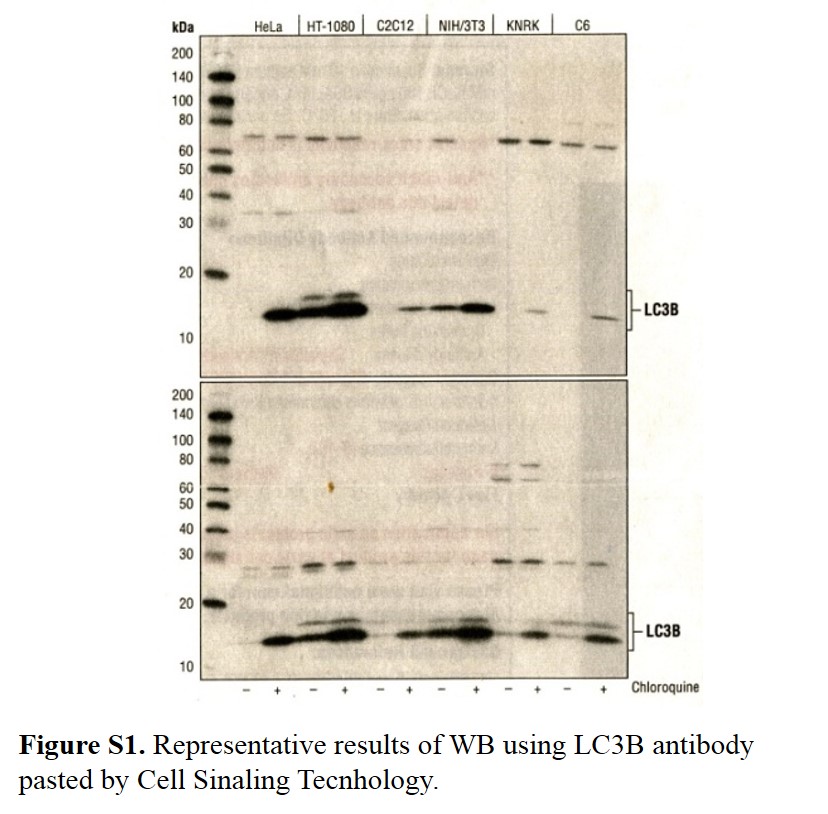

Supplement: S1 Fig — (JPG) [file pone.0183680.s001.jpg]

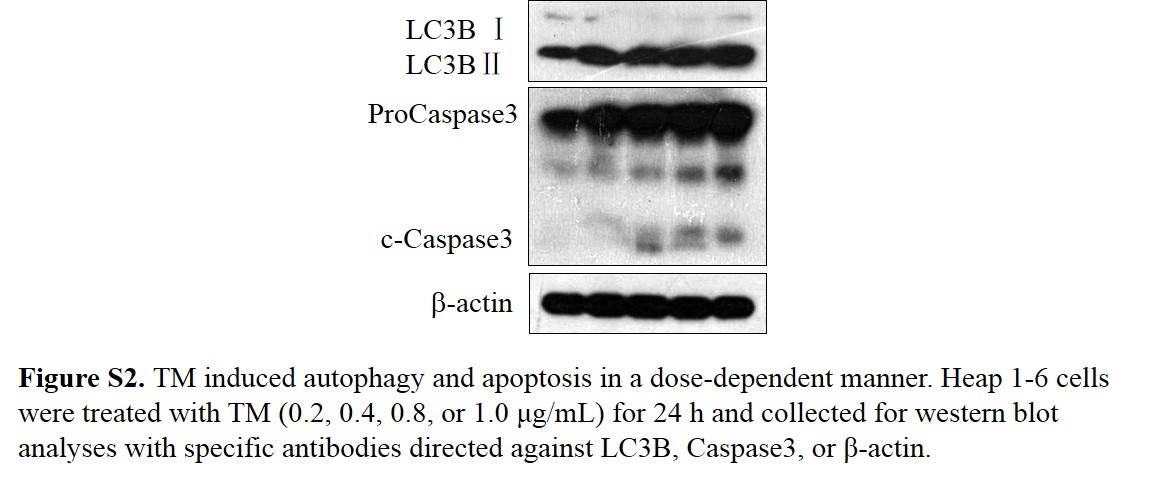

Supplement: S2 Fig — Heap 1–6 cells were treated with TM (0.2, 0.4, 0.8, or 1.0 μg/mL) for 24 h and collected for western blot analyses with specific antibodies directed against LC3B, Caspase3, or β-actin. (JPG) [file pone.0183680.s002.jpg]

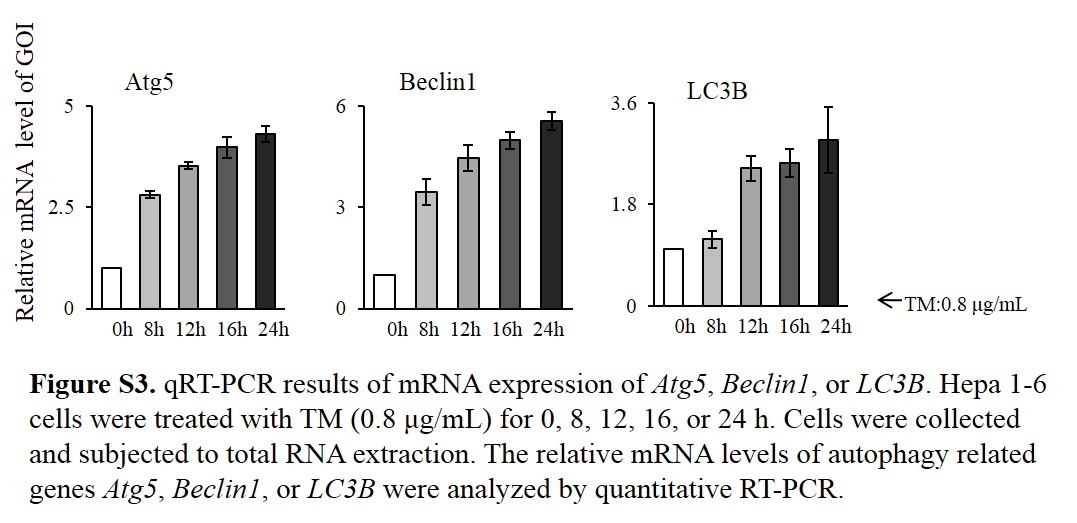

Supplement: S3 Fig — Hepa 1–6 cells were treated with TM (0.8 μg/mL) for 0, 8, 12, 16, or 24 h. Cells were collected and subjected to total RNA extraction. The relative mRNA levels of autophagy related genes Atg5, Beclin1, or LC3B were analyzed by quantitative RT-PCR. (JPG) [file pone.0183680.s003.jpg]

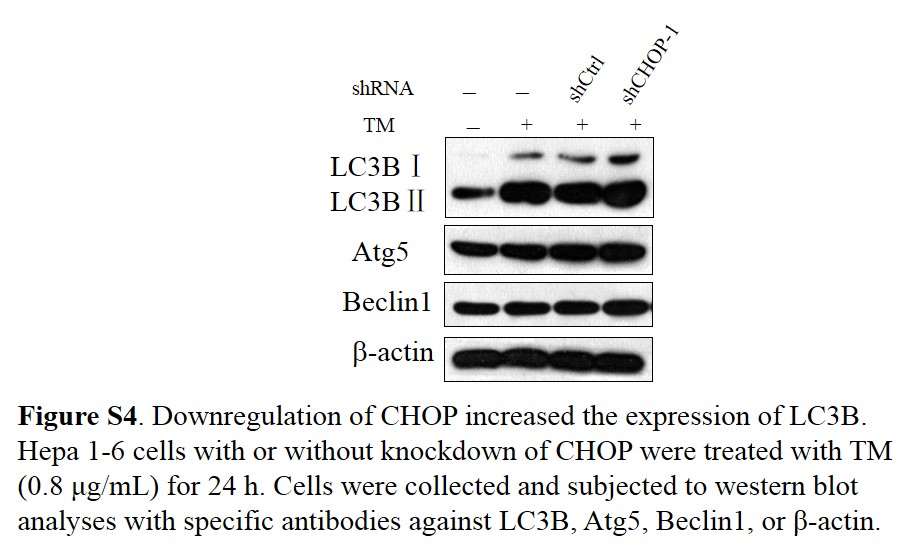

Supplement: S4 Fig — Hepa 1–6 cells with or without knockdown of CHOP were treated with TM (0.8 μg/mL) for 24 h. Cells were collected and subjected to western blot analyses with specific antibodies against LC3B, Atg5, Beclin1, or β-actin. (JPG) [file pone.0183680.s004.jpg]

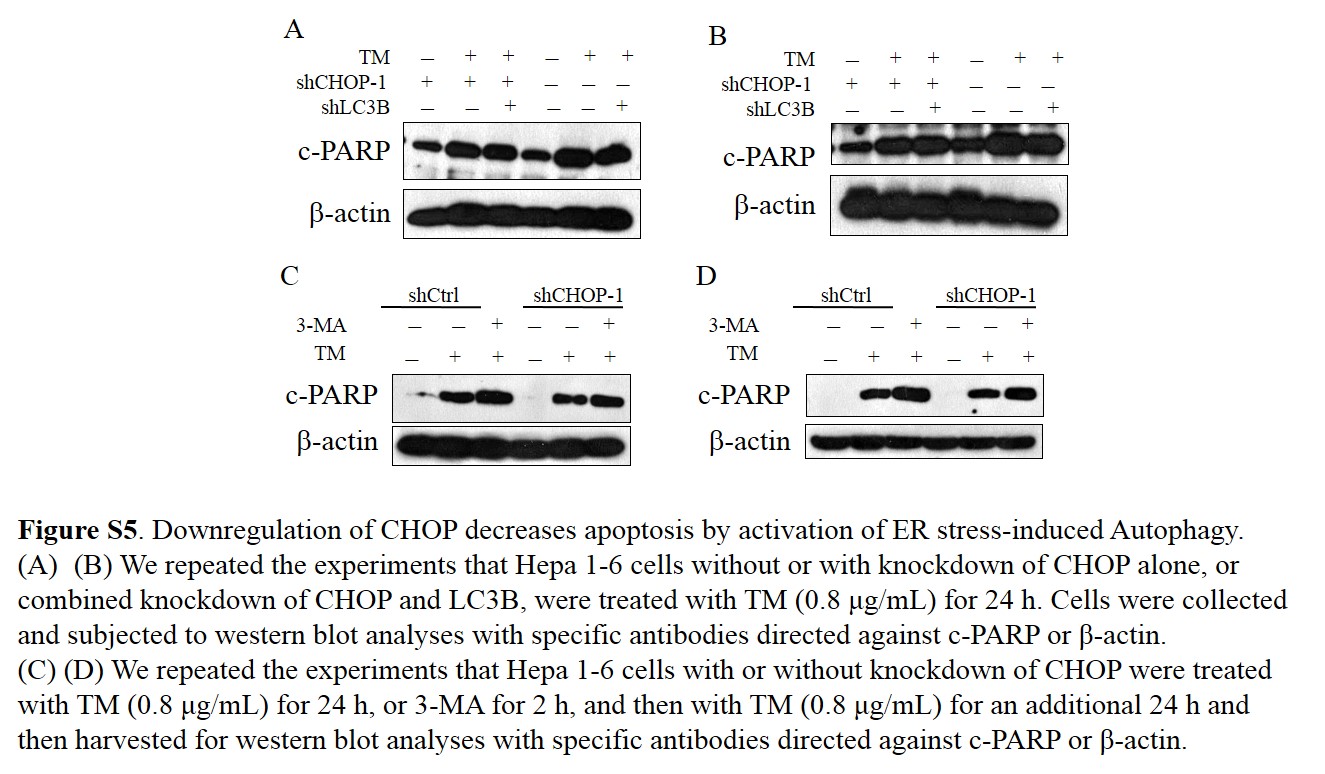

Supplement: S5 Fig — (A) (B) We repeated the experiments that Hepa 1–6 cells without or with knockdown of CHOP alone, or combined knockdown of CHOP and LC3B, were treated with TM (0.8 μg/mL) for 24 h. Cells were collected and subjected to western blot analyses with specific antibodies directed against c-PARP or β-actin. (C) (D). We repeated the experiments that Hepa 1–6 cells with or without knockdown of CHOP were treated with TM (0.8 μg/mL) for 24 h, or 3-MA for 2 h, and then with TM (0.8 μg/mL) for an additional 24 h and then harvested for western blot analyses with specific antibodies directed against c-PARP or β-actin. (JPG) [file pone.0183680.s005.jpg]

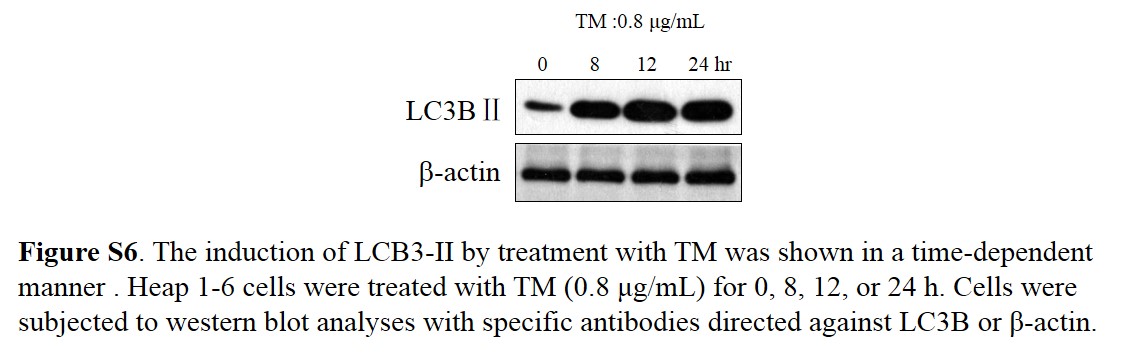

Supplement: S6 Fig — Heap 1–6 cells were treated with TM (0.8 μg/mL) for 0, 8, 12, or 24 h. Cells were subjected to western blot analyses with specific antibodies directed against LC3B or β-actin. (JPG) [file pone.0183680.s006.jpg]

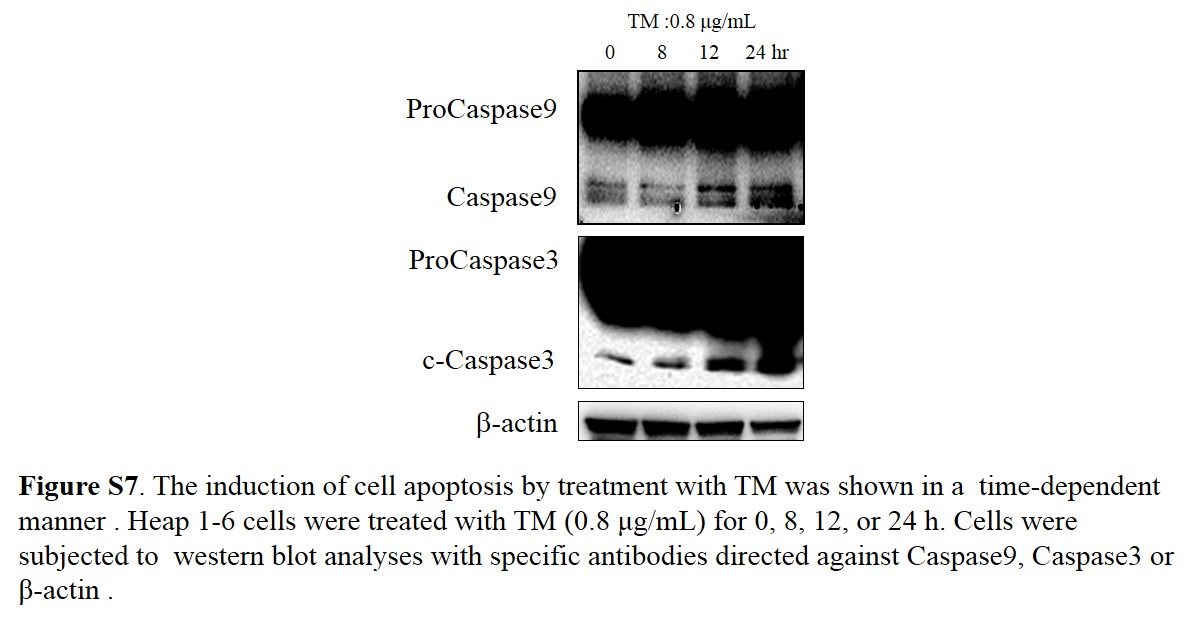

Supplement: S7 Fig — Heap 1–6 cells were treated with TM (0.8 μg/mL) for 0, 8, 12, or 24 h. Cells were subjected to western blot analyses with specific antibodies directed against Caspase9, Caspase3 or β-actin. (JPG) [file pone.0183680.s007.jpg]

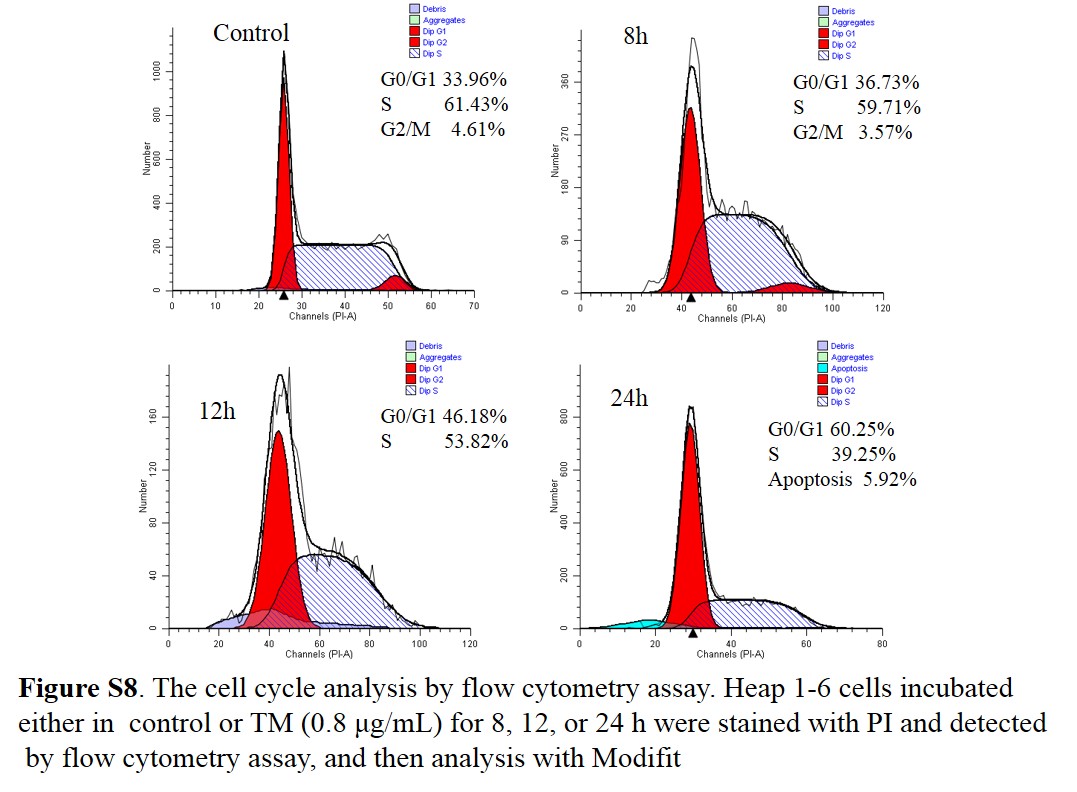

Supplement: S8 Fig — Heap 1–6 cells incubated either in control or TM (0.8 μg/mL) for 8, 12, or 24 h were stained with PI and detected by flow cytometry assay, and then analysis with Modifit. (JPG) [file pone.0183680.s008.jpg]

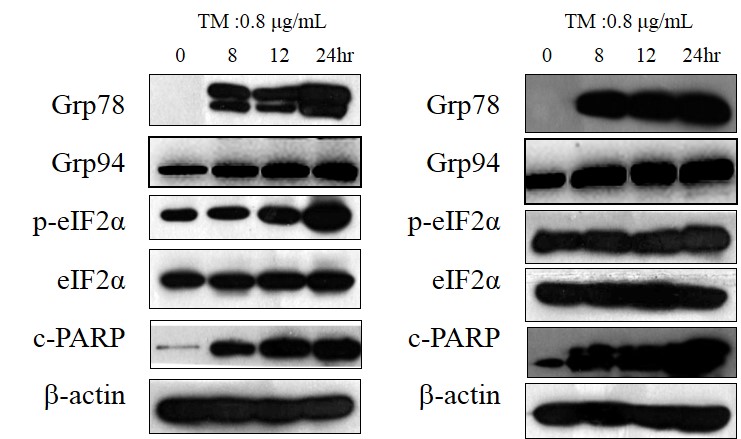

Supplement: S9 Fig — (JPG) [file pone.0183680.s009.jpg]

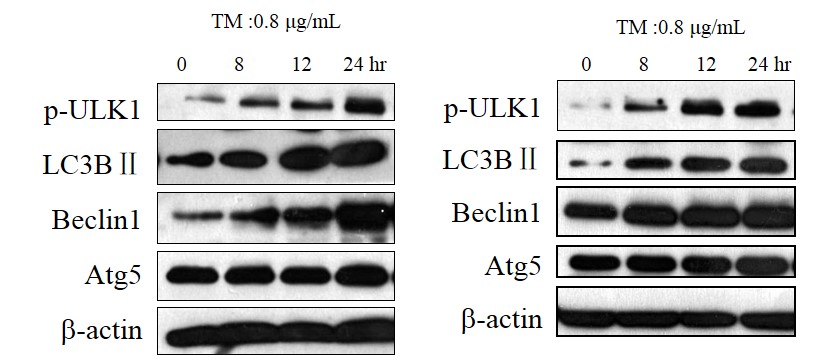

Supplement: S10 Fig — (JPG) [file pone.0183680.s010.jpg]

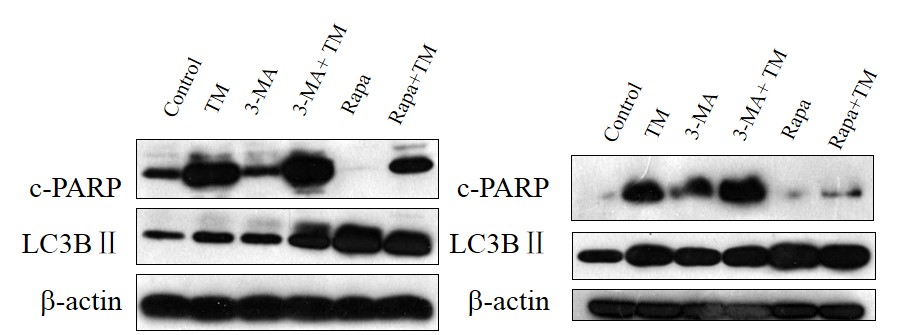

Supplement: S11 Fig — (JPG) [file pone.0183680.s011.jpg]

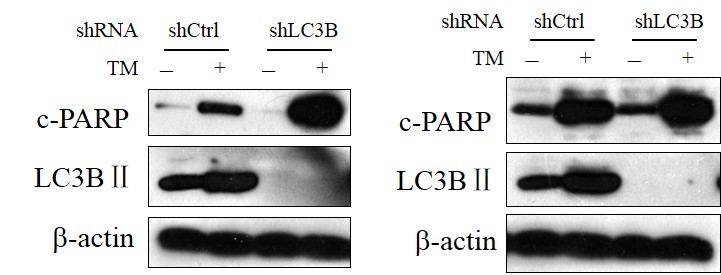

Supplement: S12 Fig — (JPG) [file pone.0183680.s012.jpg]

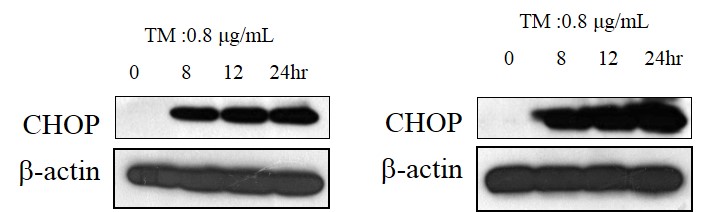

Supplement: S13 Fig — (JPG) [file pone.0183680.s013.jpg]

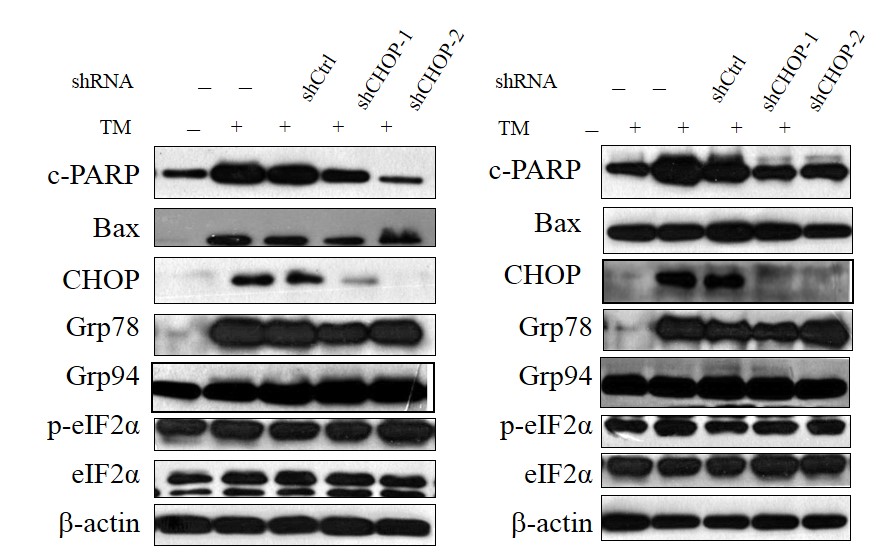

Supplement: S14 Fig — (JPG) [file pone.0183680.s014.jpg]

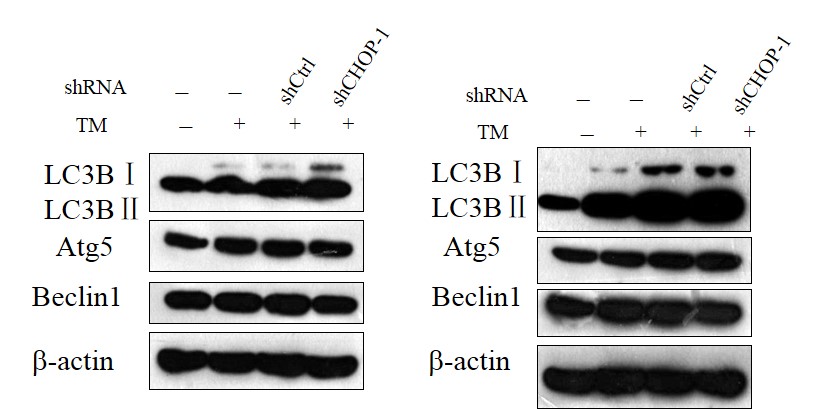

Supplement: S15 Fig — (JPG) [file pone.0183680.s015.jpg]

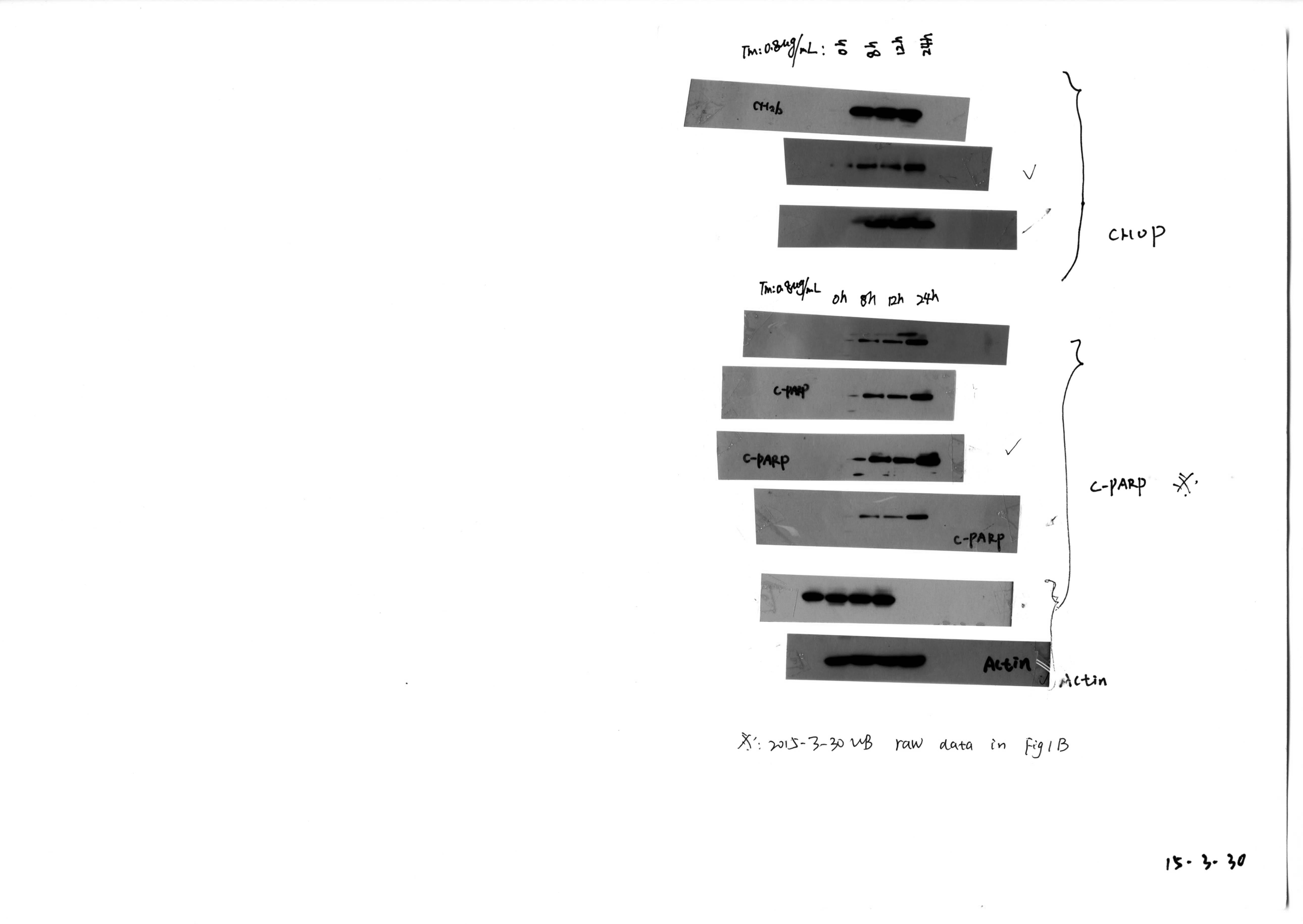

Supplement: S16 Fig — (JPG) [file pone.0183680.s016.jpg]

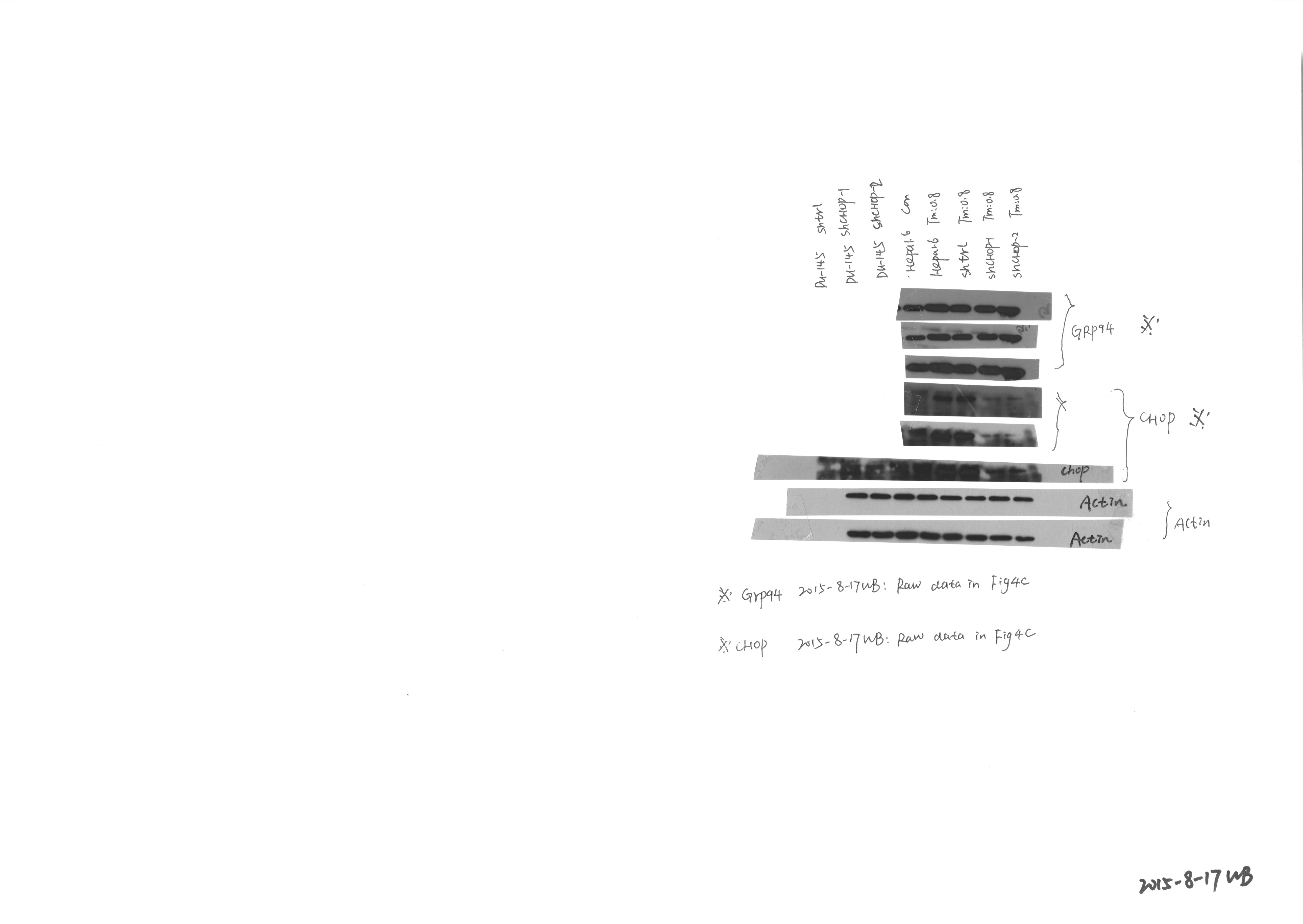

Supplement: S17 Fig — (JPG) [file pone.0183680.s017.jpg]

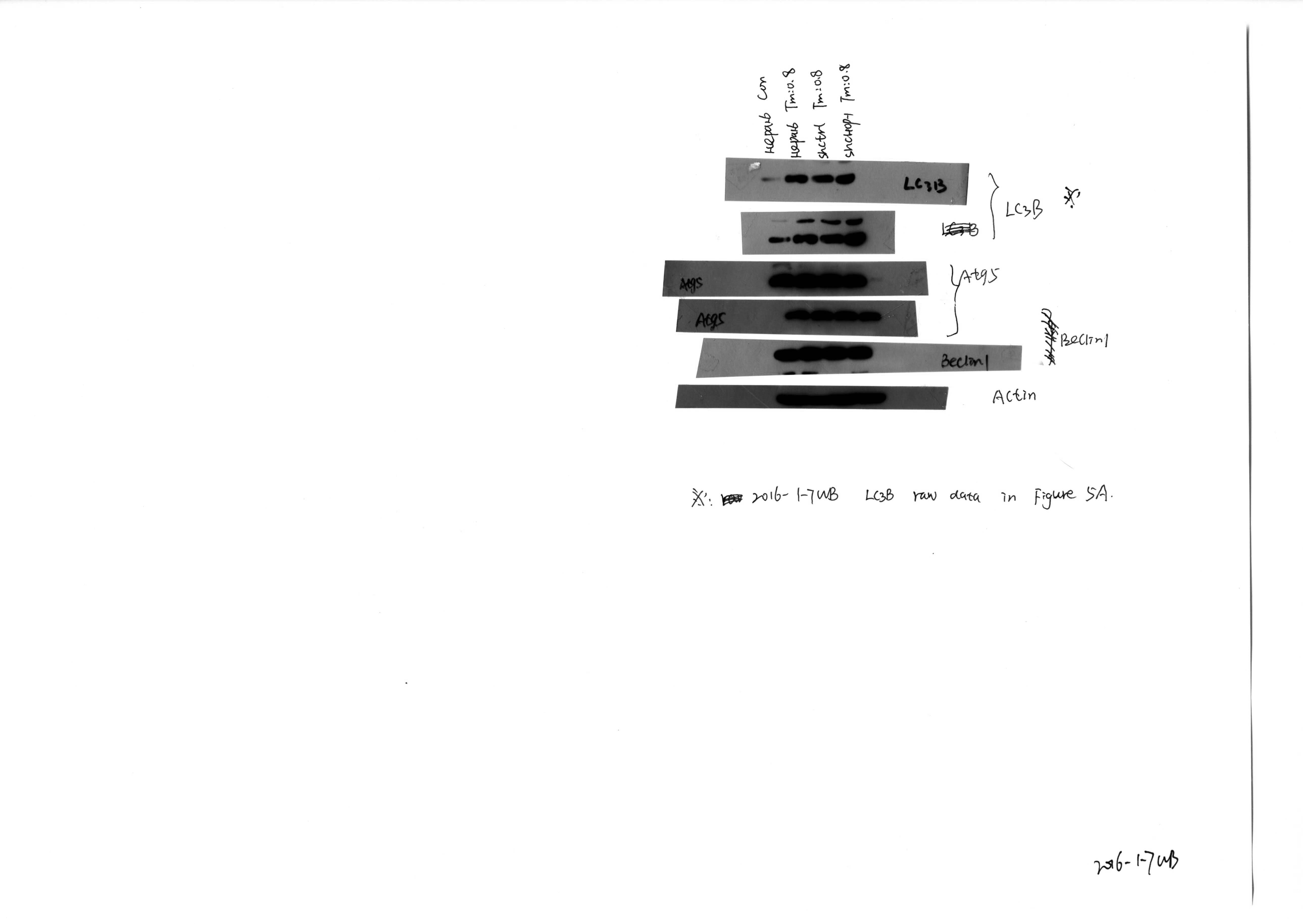

Supplement: S18 Fig — (JPG) [file pone.0183680.s018.jpg]

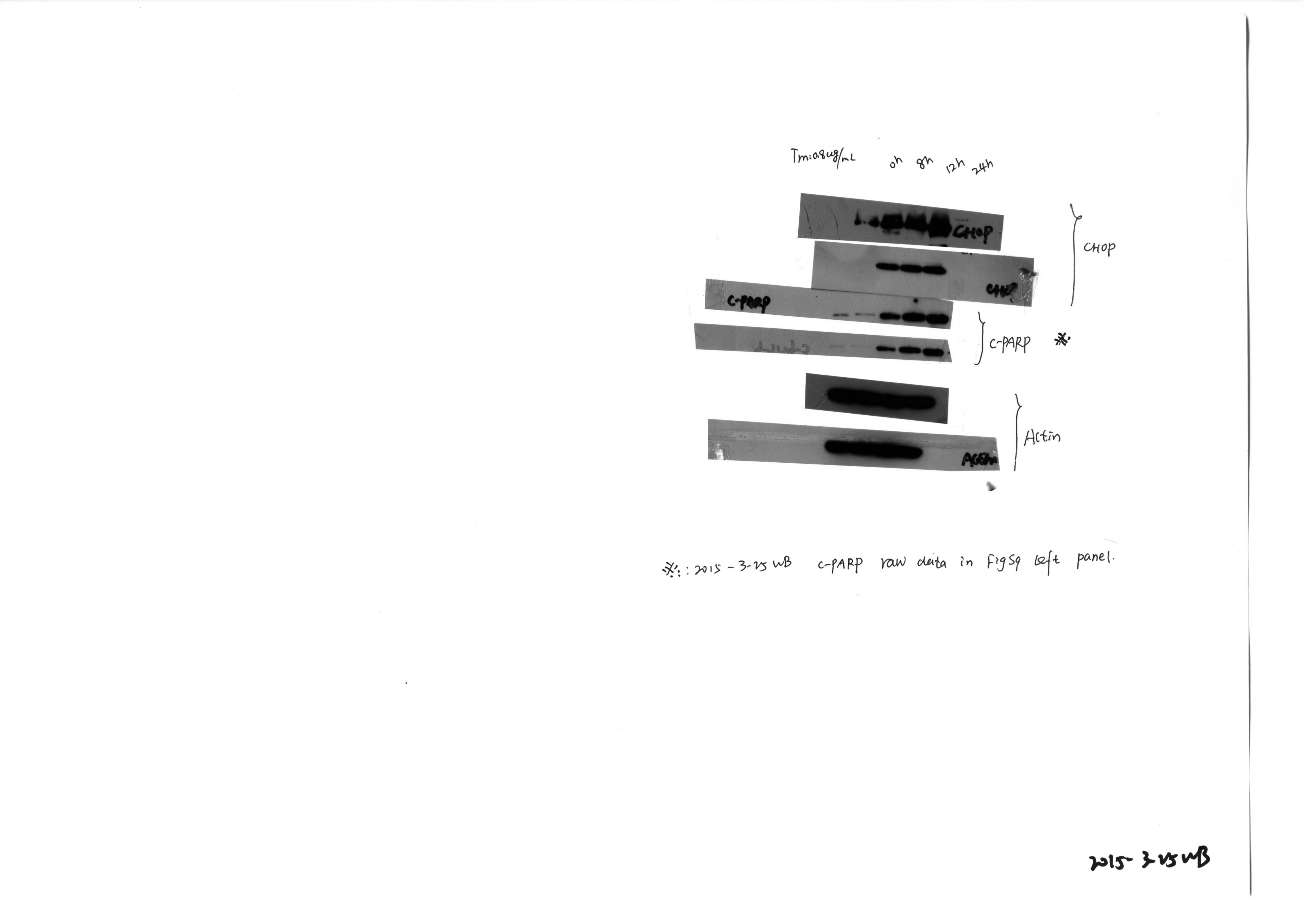

Supplement: S19 Fig — (JPG) [file pone.0183680.s019.jpg]

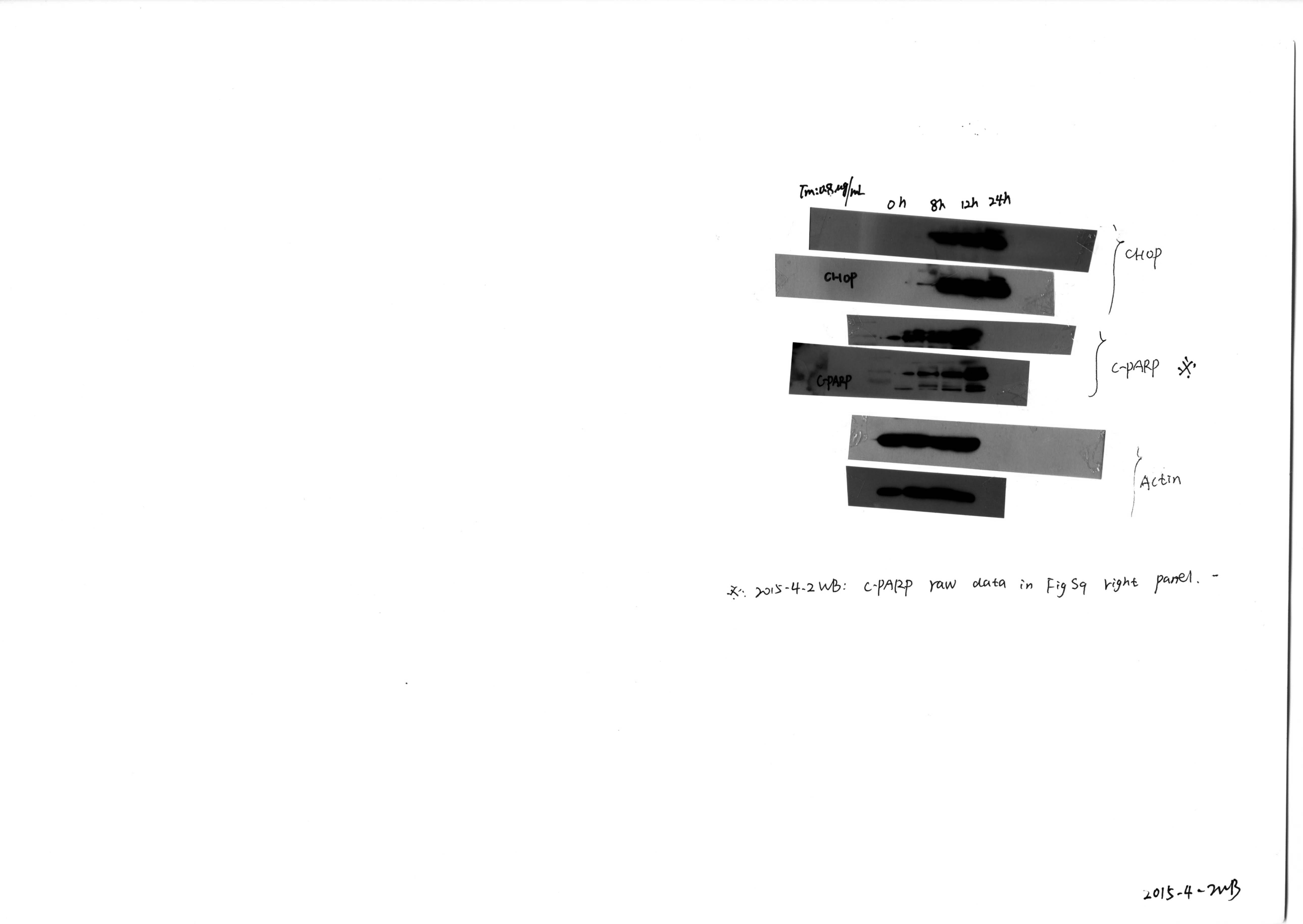

Supplement: S20 Fig — (JPG) [file pone.0183680.s020.jpg]

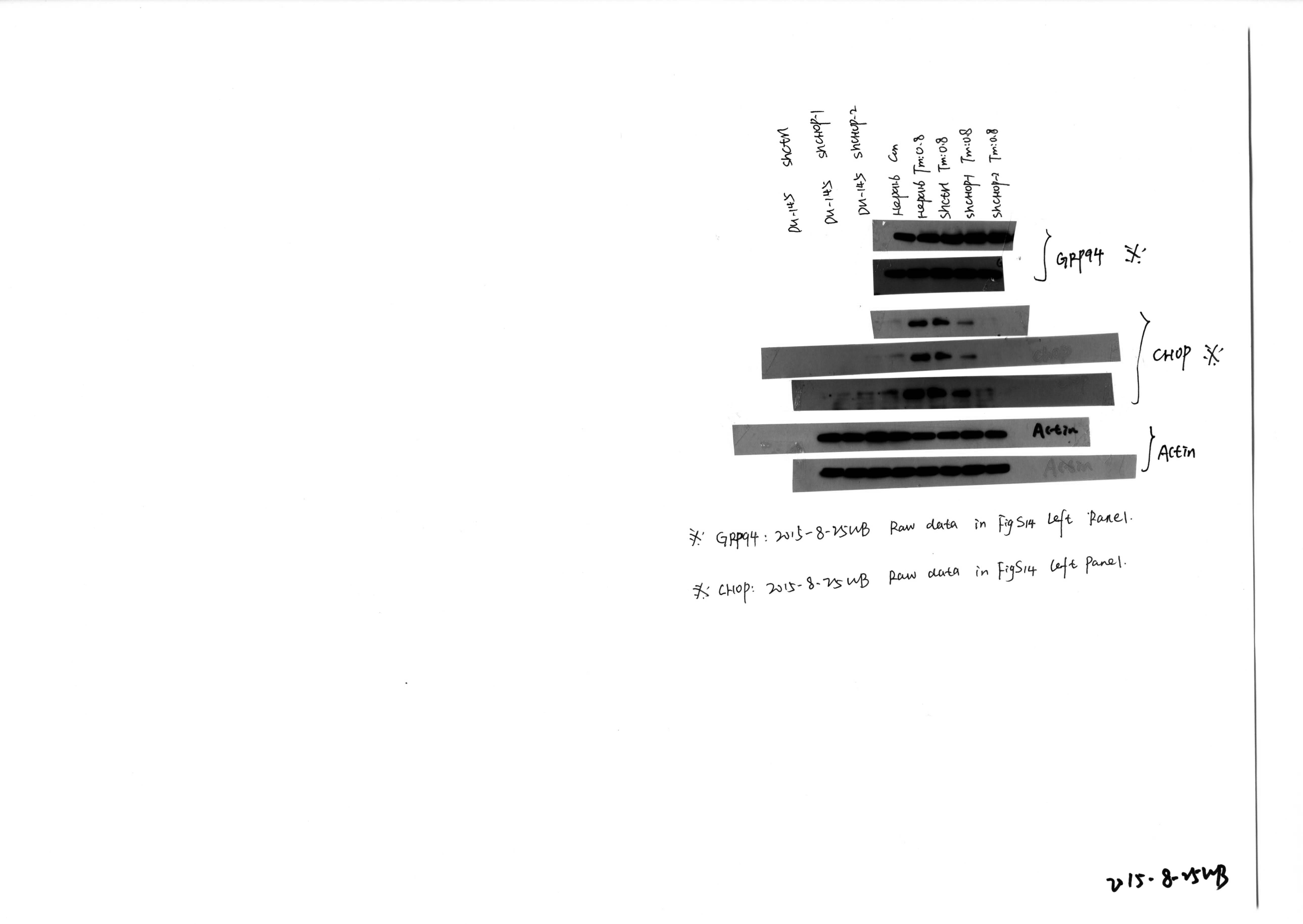

Supplement: S21 Fig — (JPG) [file pone.0183680.s021.jpg]

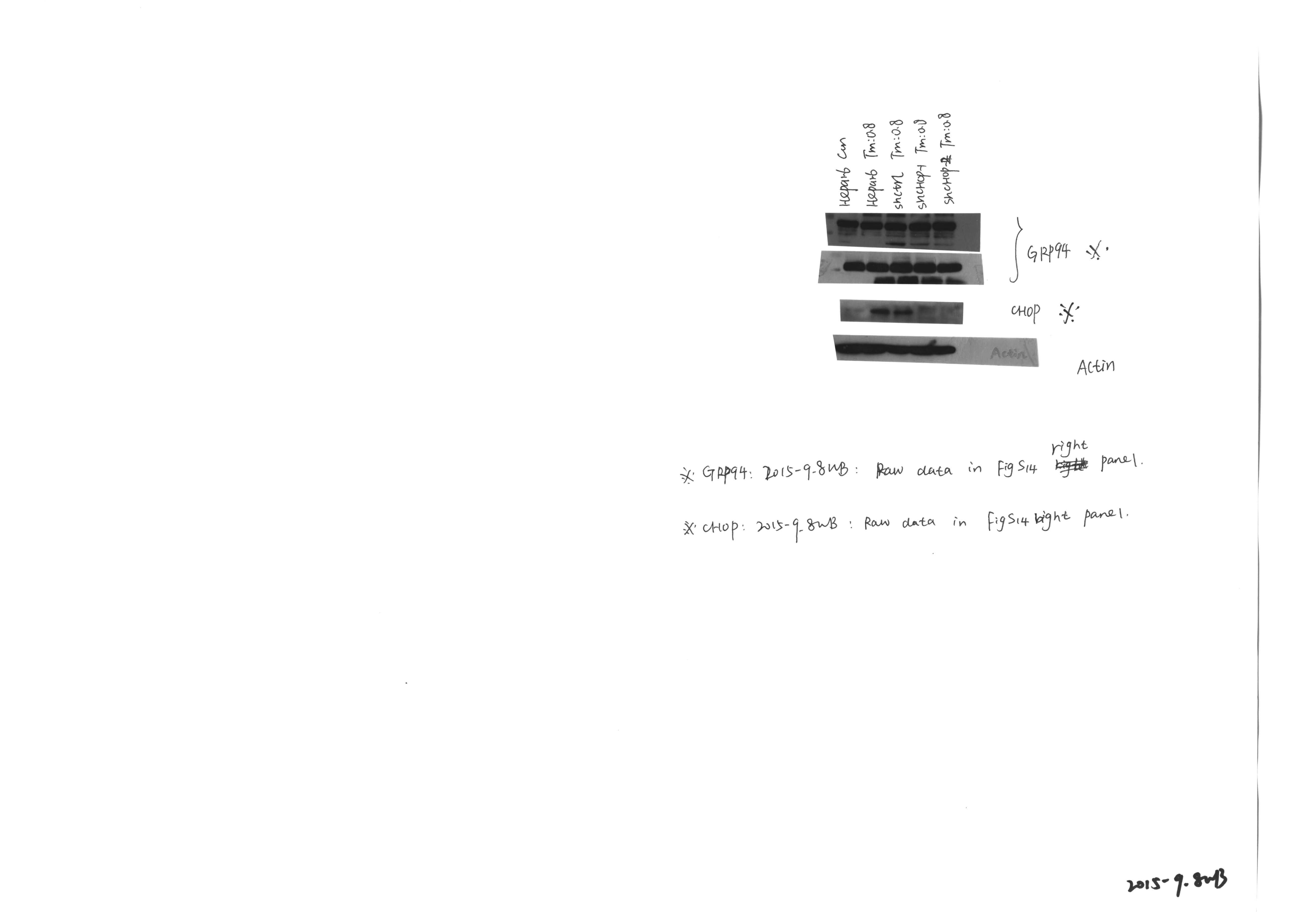

Supplement: S22 Fig — (JPG) [file pone.0183680.s022.jpg]

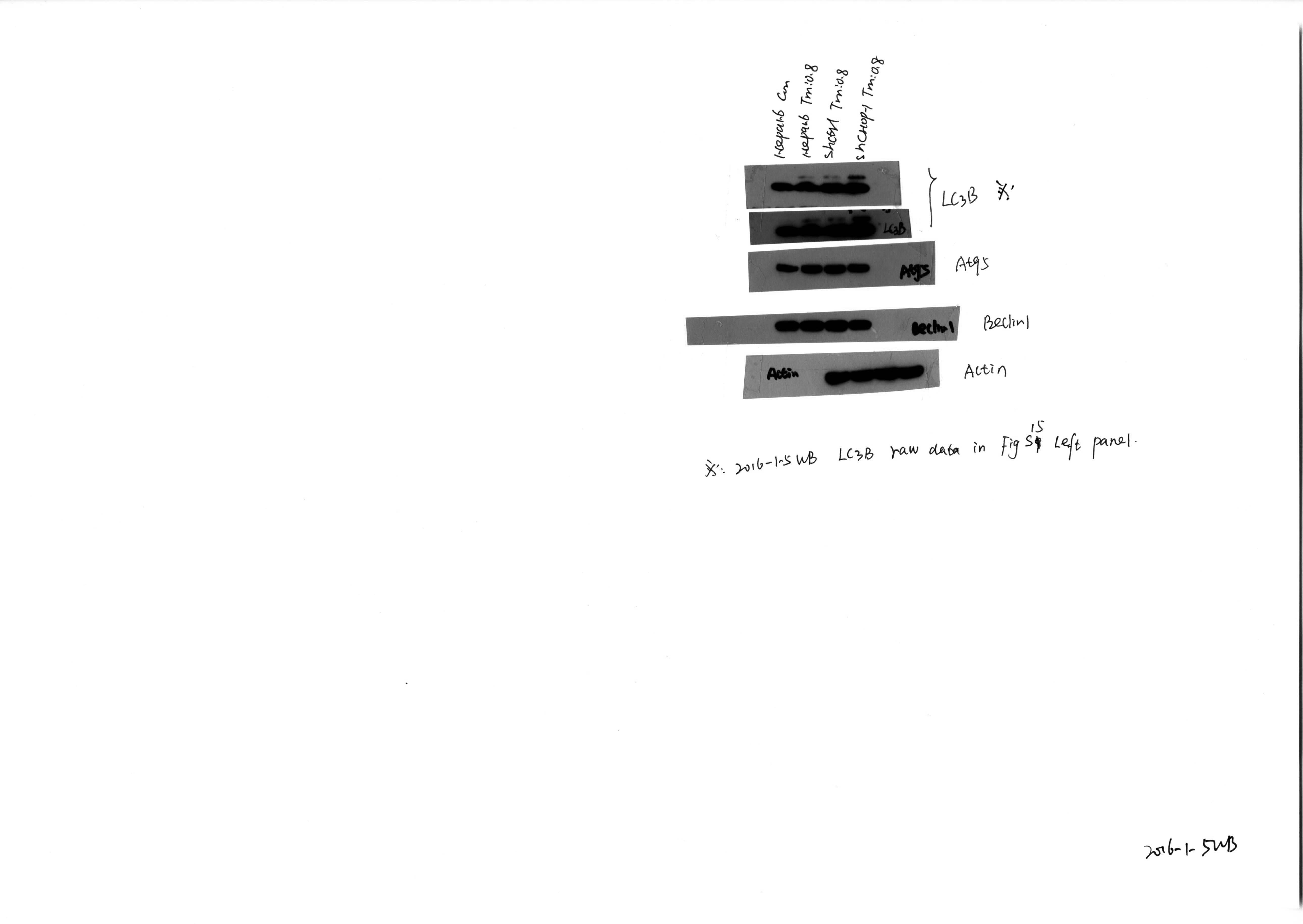

Supplement: S23 Fig — (JPG) [file pone.0183680.s023.jpg]

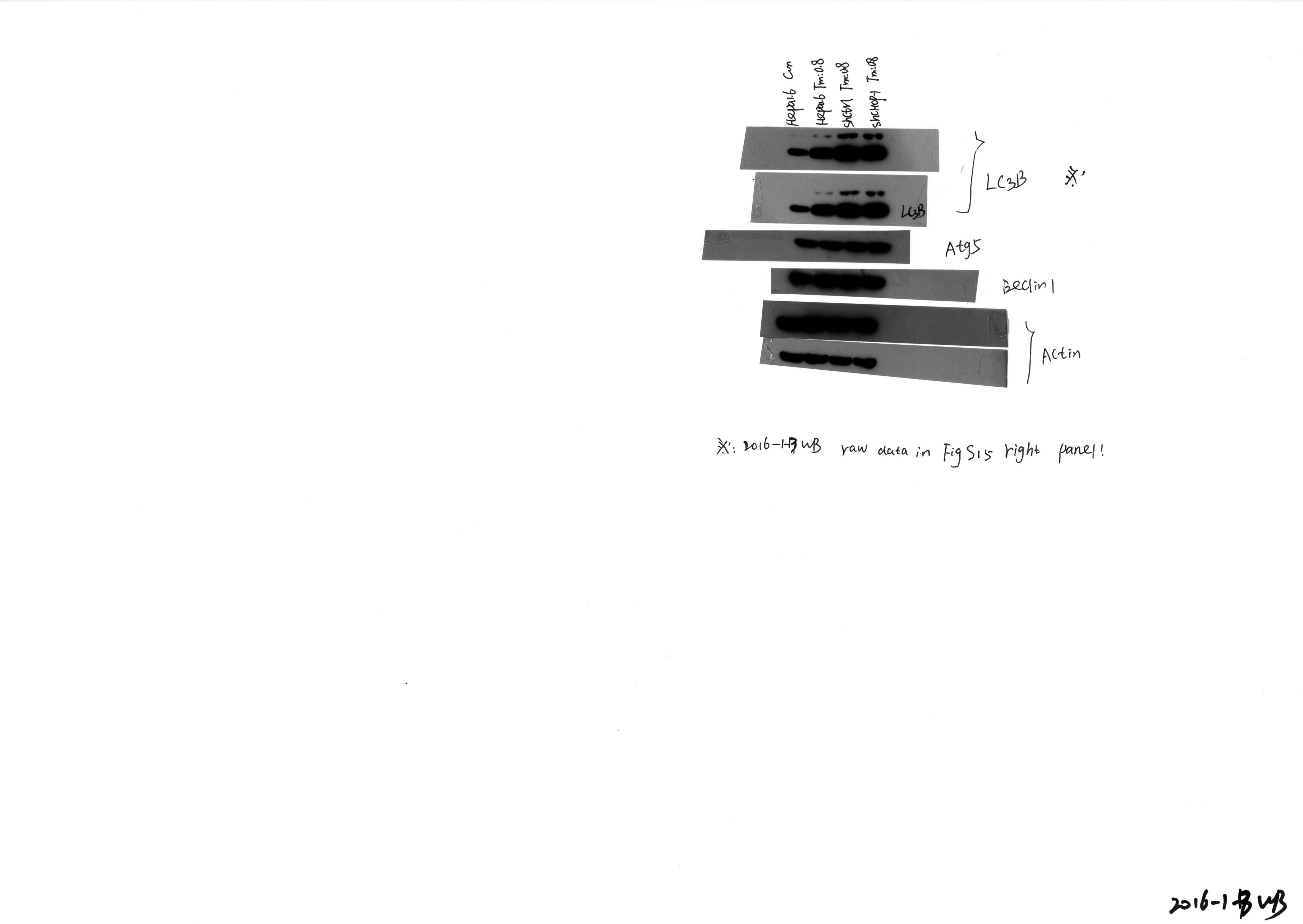

Supplement: S24 Fig — (JPG) [file pone.0183680.s024.jpg]

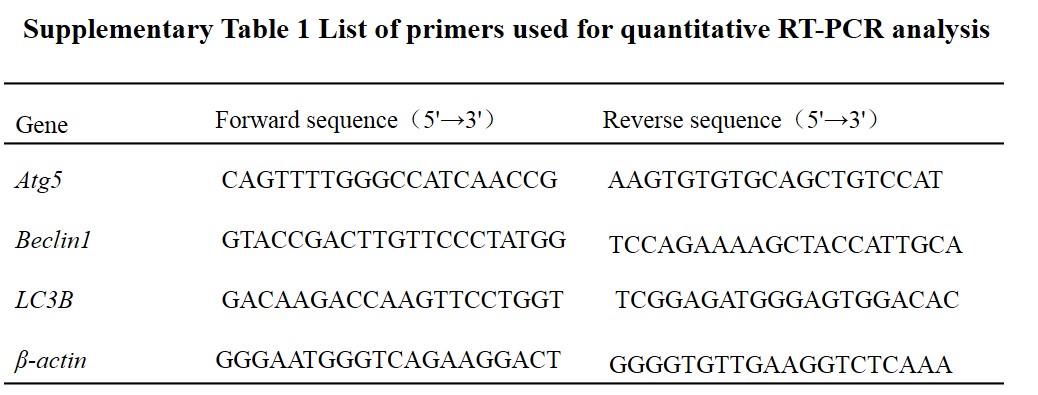

Supplement: S1 Table — (JPG) [file pone.0183680.s025.jpg]
